# Chlorophyll *a* Synthesis in *Rhodobacter sphaeroides* by Chlorophyll Synthase of *Nicotiana tabacum*

**DOI:** 10.3390/biology12040573

**Published:** 2023-04-10

**Authors:** June Kim, Jeong K. Lee, Eui-Jin Kim

**Affiliations:** 1 Department of Life Science, Sogang University, Seoul 04107, Republic of Korea; 2 Microbial Research Department, Nakdonggang National Institute of Biological Resources, Sangju 37242, Republic of Korea

**Keywords:** *Nicotiana tabacum*, ChlG, inhibition resistance, ROS, *Synechocystis* sp. PCC6803, *Rhodobacter sphaeroides*, BchG

## Abstract

**Simple Summary:**

Photosynthetic (PS) organisms utilize light energy via their PS apparatuses which contain PS pigments. Since a PS organism usually has limited wavelength regions of light absorption determined by the property of their pigments, introducing heterologous pigments can expand the light absorption regions, which could enhance the efficiency of light utilization. *Rhodobacter sphaeroides* is a bacteriochlorophyll *a* (BChl *a*)-containing PS bacterium which has been widely used in photosynthesis research and biotechnological applications. In this study, we attempted to introduce chlorophyll synthase (ChlG) into *R. sphaeroides* to synthesize chlorophyll *a* (Chl *a*) under BChl *a* formation. It was challenging because BChl *a* and its precursors can inhibit ChlG. However, ChlGs of angiosperm plants were found to possess resistance against these inhibitions. Thus, we selected ChlG of *Nicotiana tabacum*, which had the highest resistance among angiosperm ChlGs and achieved the formation of Chl *a* in the presence of BChl *a* in *R. sphaeroides* by expression of the enzyme.

**Abstract:**

The production of phytylated chlorophyll *a* (Chl *a*_P_) in *Rhodobacter sphaeroides*, which uses phytylated bacteriochlorophyll *a* (BChl *a*_P_), is the first step in expanding the light absorption spectra. Unlike the chlorophyll synthase (ChlG) of the *Synechocystis* sp. PCC6803, ChlGs of angiosperms, including *Arabidopsis thaliana*, *Nicotiana tabacum*, *Avena sativa*, and *Oryza sativa*, showed bacteriochlorophyll synthase activity and resistance to inhibition by bacteriochlorophyllide *a* (BChlide *a*), geranylgeranylated BChl *a* (BChl *a*_GG_), and BChl *a*_P_, collectively called bacteriochlorins. Among the angiosperm ChlGs, *N. tabacum* ChlG had the highest bacteriochlorophyll synthase activity and resistance to inhibition by bacteriochlorins. Expression of *N. tabacum chlG* in *R. sphaeroides* resulted in the formation of free Chl *a*_P_ in the presence of BChl *a*_P_ during photoheterotrophic growth, even though reactive oxygen species were generated.

## 1. Introduction

**Figure 1:**
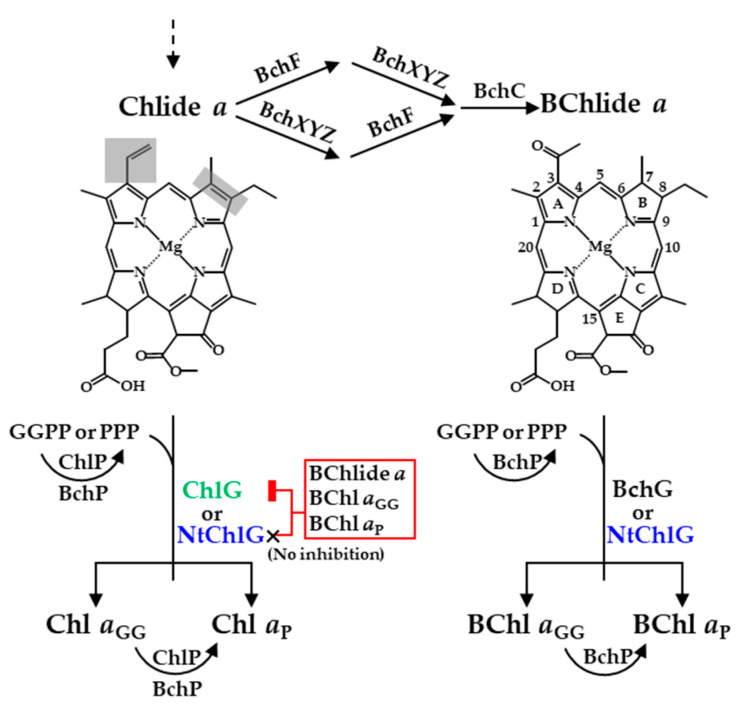
Chl *a* and BChl *a* biosynthesis in *R. sphaeroides*. Chlide *a* is metabolized by BchF and BchXYZ, and subsequently by BchC to form BChlide *a*. BChl *a*_GG_ is synthesized via the ping-pong mode by BchG using GGPP and BChlide *a* as the first and second substrates, respectively. The GG group of BChl *a*_GG_ is then reduced by BchP to yield BChl *a*_P_. Alternatively, PPP is first formed from GGPP by BchP and subsequently used as a substrate by BchG to produce BChl *a*_P_. Similarly, Chl *a*_GG_/Chl *a*_P_ are synthesized by ChlG/ChlP. ChlP activity can be substituted by BchP [8]. Generally, ChlG would be inhibited by BChlide *a*, BChl *a*_GG_, and BChl *a*_P_ (bacteriochlorin) (red box). Similarly, BchG would be inhibited by Chlide *a*, Chl *a*_GG_, and Chl *a*_P_ (collectively called chlorin). However, this study focused on the analysis of recombinant *R. sphaeroides* synthesizing more BChl *a* than Chl *a*; therefore, BchG inhibition by chlorin was negligible. *Nicotiana tabacum* ChlG (NtChlG), which is resistant to bacteriochlorin inhibition, was used to produce Chl *a*_P_ together with BChl *a*_P_ in *R. sphaeroides*. Carbon numbers and ring designations are illustrated in the BChlide *a* structure. Differences between Chlide *a* and BChlide *a* are denoted by shades in the Chlide *a* structure.

*Rhodobacter sphaeroides* is a purple anoxygenic photosynthetic (PS) bacterium that synthesizes phytylated bacteriochlorophyll *a* (BChl *a*_P_). Another group of oxygenic PS organisms, including cyanobacteria, algae, and plants, possess phytylated chlorophyll *a* (Chl *a*_P_). Chlorophyll synthase (ChlG) and bacteriochlorophyll synthase (BchG) catalyze the prenylation (esterification) of chlorophyllide *a* (Chlide *a*) and bacteriochlorophyllide *a* (BChlide *a*), respectively, with the C_20_ isoprenoid of the geranylgeranyl (GG) moiety to form geranylgeranylated Chl *a* (Chl *a*_GG_) and BChl *a* (BChl *a*_GG_) (Figure 1). The GG groups of Chl *a*_GG_ and BChl *a*_GG_ are subsequently reduced by ChlP and BchP (geranylgeranyl reductases) to form Chl *a*_P_ and BChl *a*_P_, respectively. Alternatively, phytyl pyrophosphate (PPP) is formed by the reduction in GG-pyrophosphate (GGPP) by ChlP and BchP, and PPP can be used as a substrate for ChlG and BchG [1,2] (Figure 1).

Chlide *a* and BChlide *a* are structurally similar; the differences reside in the C3 functional group and the reducing state of the C7–8 bond (Figure 1). *R. sphaeroides* BchG (RsBchG), which does not form Chl *a*, is competitively inhibited by Chlide *a* [2]. Similarly, *Synechocystis* sp. PCC6803 (hereafter *Synechocystis*) ChlG (SyChlG), which does not form BChl *a* [3], is competitively inhibited by BChlide *a* [2]. Thus, the expression of *SychlG* in *R. sphaeroides* is not likely to result in the formation of Chl *a*.

Broadening the absorption spectra of PS organisms may improve the efficiency of light utilization [4]. Recently, heterologous pigments, such as BChl *b*_P_ [5,6] and farnesylated BChl *g* (BChl *g*_F_) [7], were produced in metabolically-engineered *R. sphaeroides* under microoxic conditions. Moreover, Chl *a*_P_ was synthesized in an *R. sphaeroides* mutant, in which specific metabolic steps for BChlide *a* formation were blocked [8]. Previously, the in vitro reaction of *Avena sativa* (an angiosperm) ChlG (AsChlG) with GGPP demonstrated the prenylation of Chlide *a*, even in the presence of BChlide *a* [9], wherein BChlide *a* was also prenylated but only with poor activity. Although sequence similarities were found between SyChlG and AsChlG, the enzymes differed in their catalytic properties when BChlide *a* was used as a substrate. However, further kinetic characterization of AsChlG is required.

In this study, we aimed to produce Chl *a* in *R. sphaeroides* under BChl *a*-forming conditions via heterologous expression of *chlG*. Accordingly, ChlG must be resistant to BChl *a,* and its precursor BChlide *a*. ChlGs from angiosperms (*Arabidopsis thaliana*, *Nicotiana tabacum*, *A. sativa*, and *Oryza sativa*) and other PS organisms were analyzed to determine whether Chl *a* formation in the presence of BChl *a*/BChlide *a* is possible. Among the enzymes assessed, ChlG, with the highest resistance to bacteriochlorin inhibition, was selected to produce Chl *a* in *R. sphaeroides*.

## 2. Materials and Methods

### 2.1. Bacterial Strains and Growth Conditions

*R. sphaeroides* 2.4.1 [10] was used as the wild-type (WT) strain (Table S1) and cultured in Sistrom’s succinate-based (Sis) minimal medium [11] at 28 °C, as described previously [12]. Briefly, *R. sphaeroides* was grown aerobically with agitation at 250 rpm, and exponentially growing cells were used as the inoculum for anaerobic cultures, which were grown anaerobically in the dark in the presence of 75 mM dimethyl sulfoxide (DMSO) or photoheterotrophically at 10 W/m^2^ light. In order to alleviate reactive oxygen species (ROS) formation, photoheterotrophic growth was performed in Sis medium supplemented with 2 mM DMSO as an antioxidant [13]. *Escherichia coli* was cultured at 37 °C in Luria-Bertani (LB) medium [14] with shaking at 250 rpm. For *R. sphaeroides*, tetracycline (Tc) and kanamycin (Km) were used at a final concentration of 1 μg/mL and 25 μg/mL, respectively. For *Escherichia coli*, ampicillin (Ap), Km, and Tc were used at final concentrations of 50 μg/mL, 25 μg/mL, and 20 μg/mL, respectively.

### 2.2. Construction of Plasmids

Details of the primers and plasmids used in this study are listed in Tables S2 and S3, respectively. Amino acid sequences of BchGs/ChlGs examined in this study and detailed procedures of plasmid construction are included in the Supplementary Materials.

### 2.3. Construction of R. sphaeroides Mutants

The target gene was internally deleted using pLO1, as described previously [15]. *E. coli* S17-1 [16] was transformed using recombinant pLO1 carrying a mutated target gene insert and subsequently mobilized into *R. sphaeroides* through conjugation. Single-crossover recombinants with the Km resistant (Km^r^) phenotype were selected on Sis agar plates containing Km and were subsequently segregated on 15% (*w*/*v*) sucrose to obtain double-crossover recombinants, which were sensitive to Km. Double-crossover recombination was confirmed by genomic DNA polymerase chain reaction (PCR) analysis for the target gene.

Deletion of *bchP* in the WT and Δ*bchF* mutant (BF) [17] using pLO-bchP (Table S3) yielded the Δ*bchP* mutant (BP) (Table S1) and Δ*bchF* Δ*bchP* mutant (BFP), respectively. The subunit *bchZ* of chlorophyllide reductase (COR encoded by *bchXYZ*) in BF and BFP was interrupted using pSUPZ100 [17] to generate the Δ*bchZ* Δ*bchF* mutant (BZF1) [17] and Δ*bchZ* Δ*bchF* Δ*bchP* mutant (BZFP), respectively (Table S1).

### 2.4. Isolation and Spectral Analysis of the Membrane Fraction from R. sphaeroides

Cells were harvested and resuspended in 10 mM phosphate-buffered saline (PBS) at pH 7.4, followed by lysis using a high-pressure homogenizer (Microfluidizer^TM^, Microfluidics Corporation, Newton, MA, USA) at 20,000 psi. The homogenate was centrifuged at 6000× *g* at 4 °C for 10 min, and the supernatant was subjected to ultracentrifugation at 150,000× *g* at 4 °C for 1 h. The supernatant, after ultracentrifugation, was decanted as a cytosolic fraction. Membranes in the pellet were solubilized in PBS supplemented with 1% (*w*/*v*) *n*-dodecyl-β-D-maltoside (DDM), followed by continuous mixing at 4 °C for 1 h. Insoluble material was removed by centrifugation at 12,000× *g* at 4 °C for 5 min. The total protein in the membrane fraction was determined using the Lowry method [18]. Before absorption spectra analysis using a UV-Vis spectrophotometer (UV 2550; Shimadzu, Kyoto, Japan), the samples were adjusted to the same protein level.

### 2.5. Pigment Extraction from R. sphaeroides

Pigments were extracted from cells using an acetone/methanol (7/2, *v/v*) mixture. The sample was vortexed vigorously for 1 min and centrifuged at 14,000× *g* at 4 °C for 5 min. The supernatant was filtered using a syringe filter (0.45 μm pore size, Whatman, Maidstone, UK), and the filtrate was subjected to HPLC analysis. The HPLC system was composed of an LC-6AD dual pump (Shimadzu, Japan) equipped with a Zorbax ODS column (Agilent Technologies, Santa Clara, CA, USA; particle size, 5 μm; diameter × length, 4.6 × 250 mm), a fluorescence detector, and a UV-Vis absorbance detector.

### 2.6. Preparation of BChlide a, Chlide a, BChl a_GG_, and Chl a_GG_

BChlide *a* and Chlide *a* were prepared from BChl *a*_P_ and Chl *a*_P_ (Sigma-Aldrich, St. Louis, MO, USA), respectively, using chlorophyllase of *A. thaliana* (AtChlase). C-terminally strep-tagged AtChlase was purified from *E. coli* BL21 (DE3) (Stratagene, San Diego, CA, USA) (Table S1) transformed with pChlase (Table S3). Cells were cultured until the OD_600_ reached 0.6, and subsequently, anhydrotetracycline (Sigma-Aldrich) was added at a final concentration of 0.2 μg/mL. Cells were incubated for another 12 h and harvested by centrifugation at 6000× *g* at 4 °C for 5 min. The cell pellet was resuspended in Buffer A (50 mM Tris-Cl, pH 8.0, 150 mM NaCl), and cells were lysed via sonication on ice for 5 min for a total of three times. The sample was centrifuged at 8000× *g* at 4 °C for 15 min, and the supernatant was loaded onto Strep-Tactin^TM^ resin (IBA Life Sciences, Göttingen, Germany). The resin was washed with Buffer A, and the AtChlase was eluted with Buffer A containing 2.5 mM desthiobiotin.

The chlorophyllase reaction was performed as previously described [3]. BChlide *a* and Chlide *a* were quantified using the extinction coefficients 42.1 mM^−1^ cm^−1^ at 773 nm and 54.1 mM^−1^ cm^−1^ at 665 nm, respectively [19].

BChl *a*_GG_ was prepared from BP (Table S1), and Chl *a*_GG_ was prepared from BZFP-WSCP-Ntc (Table S1), similar to the method used in a previous study [8]. The cells were grown anaerobically in Sis medium supplemented with 75 mM DMSO in the dark. BChl *a*_GG_ and Chl *a*_GG_ were extracted using dioxane and purified via Sepharose CL-6B column chromatography, as described previously [20].

The extinction coefficients of BChl *a*_GG_ and Chl *a*_GG_ were similar to those of BChl *a*_P_ and Chl *a*_P_, respectively. Equivalent levels of BChlide *a* were produced when either BChl *a*_GG_ or BChl *a*_P_ at the same absorbance were digested with AtChlase, and similar results for Chlide *a* were obtained with Chl *a*_GG_ and Chl *a*_P_. The molar extinction coefficients of BChl *a*_GG_ and Chl *a*_GG_ were estimated to be 83.9 mM^−1^ cm^−1^ at 771 nm and 85.6 mM^−1^ cm^−1^ at 662 nm [19], respectively.

### 2.7. HPLC Determination of BChl a_GG_, Chl a_GG_, BChl a_P_, and Chl a_P_

BChl *a*_GG_, Chl *a*_GG_, BChl *a*_P_, and Chl *a*_P_ were detected by HPLC as described previously [3], with minor modifications. Samples were injected into a 200 μL injection loop and eluted using linear gradients of acetone/water mixture for 29 min at a flow rate of 1 mL/min. Solvent compositions were as follows: 0 min, 70% acetone; 2 min, 70% acetone; 4 min, 82% acetone; 15 min, 88% acetone; 19 min, 100% acetone; 24 min, 100% acetone; and 29 min, 70% acetone. Quantification of BChl *a*_GG_ and BChl *a*_P_ was performed by monitoring the absorbance at 771 nm, and Chl *a*_GG_ and Chl *a*_P_ were evaluated at 662 nm. Commercial BChl *a*_P_ and Chl *a*_P_ (Sigma-Aldrich) were used to generate standard curves for the quantification of BChl *a*_GG_ (BChl *a*_P_) and Chl *a*_GG_ (Chl *a*_P_), respectively.

### 2.8. Heterologous Expression and Quantification of BchG and ChlG in E. coli

*E. coli* BL21 (DE3) transformed with pET-Rsb, pET-Syc, pET-Atc, pET-Ntc, pET-Asc, and pET-Osc (Table S3) were cultured at 37 °C in 30 mL LB medium containing Km until OD_600_ reached 0.6. Then, 1 mM isopropyl *β*-D-1-thiogalactopyranoside (IPTG) was added, and cells were further incubated at the same temperature for 6 h. Cells were harvested by centrifugation at 4000× *g* and 4 °C for 5 min and washed once with PBS. Cell pellets were resuspended in 5 mL of Buffer B (120 mM potassium acetate, 10 mM magnesium acetate, 50 mM HEPES-KOH, pH 7.6, 14 mM mercaptoethanol, and 10% glycerol) [3], followed by sonication on ice for 5 min for a total of three times. The resulting sample was centrifuged at 8000× *g* and 4 °C for 5 min, and the supernatant (cell lysate) was used as the enzyme source.

Since BchG and ChlGs are translationally fused to His_6_-tag at C-termini (RsBchG, 34.0 kDa; SyChlG, 36.6 kDa; AtChlG (*A. thaliana* ChlG), 36.9 kDa; NtChlG (*N. tabacum* ChlG), 37.0 kDa; AsChlG, 37.4 kDa; OsChlG (*O. sativa* ChlG), 37.0 kDa), their levels in *E. coli* lysates were determined by western immunoblot analysis using an anti-His_6_-tag antibody (MBL, Woburn, MA, USA). A standard curve for quantification was prepared as described previously [21]. Cell lysates containing BchG-His_6_ or ChlG-His_6_ were loaded on SDS-polyacrylamide (12%) gel in parallel with C-terminally His_6_-tagged protoporphyrin ferrochelatase of *Vibrio vulnificus* (PpfC, 38.0 kDa) [22] as a quantitative standard. The protein bands of BchG-His_6_ and ChlG-His_6_ were quantified by densitometry using Image J [23] after western immunoblot analyses.

### 2.9. Kinetic Analysis of BchG and ChlG Activities

BchG and ChlG activities were determined as described previously [3]. Cell lysates (1 mg protein) were mixed with 200 μL of Buffer B supplemented with 0.5 mM ATP, 50 μM GGPP (or 50 μM PPP), and varying concentrations of BChlide *a* (10–80 μM) for BchG activity assay or Chlide *a* (5–40 μM) for ChlG activity assay. The reaction mixture was incubated at 30 °C for 3 h for BchG activity and for 30 min for ChlG activity. Inhibitions of BchG and ChlGs were analyzed in the presence of inhibitors BChlide *a*/Chlide *a*, BChl *a*_GG/_ Chl *a*_GG_, and BChl *a*_P_/Chl *a*_P_ at 10, 20, and 40 μM, respectively.

The reaction was stopped by adding 800 μL acetone, and the mixture was centrifuged at 12,000× *g* and 4 °C for 5 min. The supernatant was filtered and injected into the HPLC system, and BChl *a* and Chl *a* were determined as described earlier. Kinetic parameters, including inhibition constant, were calculated from the data sets of initial reaction rate *V*_0_ by nonlinear regression fitting to competitive inhibition model using SigmaPlot ver. 14 (Systat Software, San Jose, CA, USA). All measurements were independently repeated three times, and data are shown as the mean ± standard deviation (SD).

### 2.10. Determination of Intracellular ROS Levels

ROS levels were determined as described previously [24]. Cell culture aliquots were centrifuged at 12,000× *g* and 4 °C for 1 min, and the pellet was resuspended in Sis medium. The oxidation-sensitive fluorescent probe, 2,7-dihydrodichlorofluorescein diacetate (H_2_DCFDA; Sigma-Aldrich), was added to the cell suspension at a final concentration of 10 μM. Samples were then incubated at 30 °C for 30 min, and fluorescence was measured at 525 nm (after excitation at 492 nm) using a microplate reader (EnSpire; PerkinElmer, Waltham, MA, USA). Samples were evaluated in triplicate, and the controls were not treated with H_2_DCFDA. All measurements were performed independently in triplicates, and the data are presented as the mean ± SD.

Singlet oxygen levels were determined using Singlet Oxygen Sensor Green (SOSG, Invitrogen, Waltham, MA, USA). Cells were grown aerobically, and aliquots (5% volume ratio) of cells in the exponential phase were inoculated into a fresh Sis medium (supplemented with 50 μM SOSG) at a final OD_660_ of 0.1. Cells were grown photoheterotrophically for 1 h, and the fluorescence of reacted SOSG at 525 nm was evaluated after excitation at 504 nm. Cells grown in the dark for the same period were used as controls. Quantification was performed independently in triplicates, and the data are presented as mean ± SD.

### 2.11. Statistical Analysis

All results are presented as mean ± SD. In order to assess differences between the two samples, the Student’s *t*-test was applied to the statistical analysis, in which significant differences were annotated by the following symbols: ns, *p* > 0.05; *, *p* ≤ 0.05; **, *p* ≤ 0.01; ***, *p* ≤ 0.001.

## 3. Results

### 3.1. Angiosperm ChlGs Prenylated BChlide a

**Figure 2:**
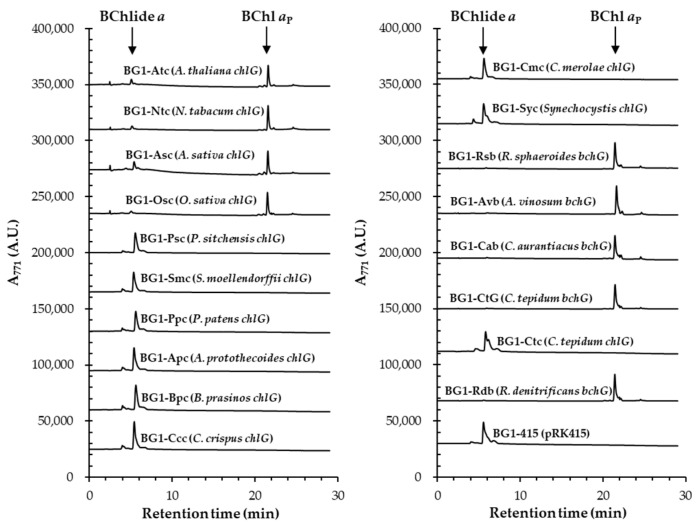
Formation of BChl *a*_P_ in BG1 expressing *bchG*s and *chlG*s from various PS organisms. BG1 cells expressing *bchG*s and *chlG*s were cultured in the dark with 75 mM DMSO for seven days. Total pigments were extracted from the cells and subjected to HPLC analysis. BChlide *a* and BChl *a*_P_ are indicated by arrows, which were detected by absorbance at 771 nm. A.U., Arbitrary Unit.

**Figure 3:**
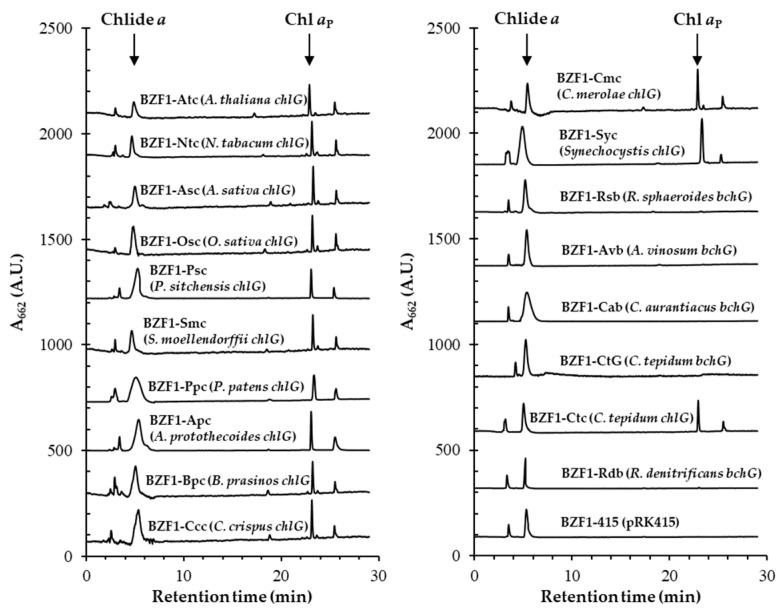
Formation of Chl *a*_P_ in BZF1 expressing *bchG*s and *chlG*s from various PS organisms. BZF1 cells expressing *bchG*s and *chlG*s were cultured in the dark with 75 mM DMSO for seven days. Total pigments were extracted from the cells and subjected to HPLC analysis. Chlide *a* and Chl *a*_P_ are indicated by arrows, which were detected by absorbance at 662 nm. A.U., Arbitrary Unit.

As AsChlG formed BChl *a*_GG_ and also synthesized Chl *a*_GG_ in vitro in the presence of BChlide *a* [9], we examined whether ChlGs from other PS organisms possess such broad substrate specificities. A *bchG*-deleted mutant (BG1) [2] and a *bchZ*/*bchF*-deleted mutant (BZF1) [17] of *R. sphaeroides* accumulated BChlide *a,* and Chlide *a* in cells (Figure 1), and these mutants could be used to examine BchG and ChlG activity, respectively. BG1 and BZF1 were transformed to express *bchG*s/*chlG*s from diverse PS organisms (Table S1). The recombinant strains were cultured anaerobically in the dark condition with DMSO (75 mM), and extracted pigments were analyzed by HPLC to assess the formation of intracellular BChl *a*_P_ or Chl *a*_P_. BG1 transformants carrying *bchG*s of PS bacteria (purple sulfur, *Allochromatium vinosum*; green nonsulfur, *Chloroflexus aurantiacus*; green sulfur, *Chlorobaculum tepidum*; and the aerobic anoxygenic PS bacterium, *Roseobacter denitrificans*) synthesized BChl *a*_P_ as BG1 carrying *R. sphaeroides bchG* (BG1-Rsb) did (Figure 2). Surprisingly, BG1 carrying *chlG*s of angiosperm (*A. thaliana*, *N. tabacum*, *A. sativa*, and *O. sativa*) also synthesized BChl *a*_P_, whereas BG1 carrying other *chlG*s (vascular plants, *Picea sitchensis* (gymnosperm) and *Selaginella moellendorffii* (lycophyte); moss, *Physcomitrella patens*; algae, *Auxenochlorella protothecoides* and *Bathycoccus prasinos* (green algae), *Chondrus crispus* and *Cyanidioschyzon merolae* (red algae); cyanobacterium, *Synechocystis*; green sulfur bacterium, *C. tepidum*) failed (Figure 2). In contrast, all BZF1 transformants carrying *chlG*s synthesized Chl *a*_P_, while all BZF1 carrying *bchG*s failed (Figure 3). Another homolog of *NtchlG* (named *NtchlG2* hereafter) was found in the genome of *N. tabacum*, which showed 99% similarity (99% identity) to NtChlG. The formation of BChl *a*_P_ by NtChlG2 was similar to that of NtChlG. (Table S4). The results are summarized in Table 1. Thus, angiosperm ChlGs were confirmed to possess both ChlG and BchG activity, whereas other BchGs and ChlGs only possess either of the activities (Table 1).

**Figure 4:**
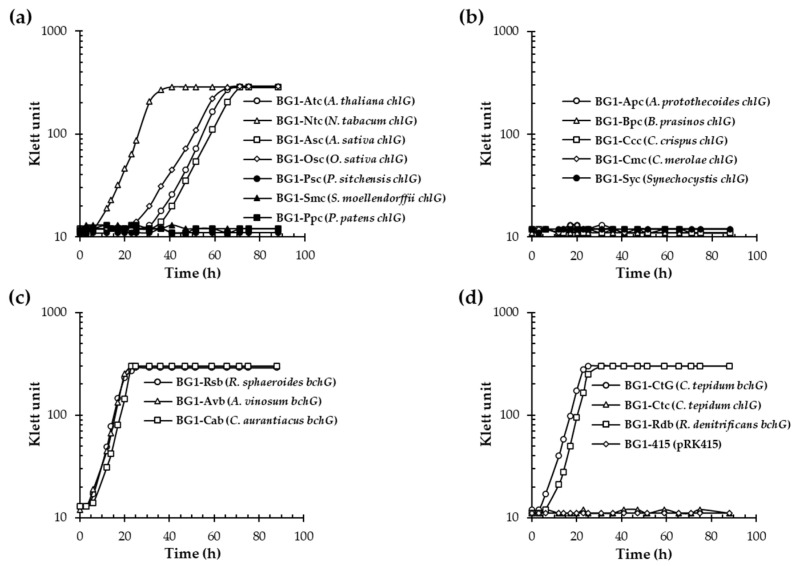
Photoheterotrophic growth of BG1 expressing *bchG*s and *chlG*s from various PS organisms. The growths of BG1 expressing *chlG* of plants (**a**), *chlG* of algae and cyanobacterium (**b**), and *bchG*/*chlG* of PS bacteria (**c**,**d**) were recorded. BG1-Rsb and BG1-415 were included as controls.

BG1 is incompetent for PS growth because it does not synthesize BChl *a*_P_ while accumulating BChlide *a*. Only an enzyme able to prenylate the BChlide *a* to BChl *a* can restore the PS growth [25]. The BchGs of PS bacteria (*A. vinosum*, *C. aurantiacus*, *C. tepidum*, and *R. denitrificans*) restored the photoheterotrophic growth of BG1-like BG1-Rsb (Figure 4c,d). ChlGs of angiosperm (*A. thaliana*, *N. tabacum*, *A. sativa*, and *O. sativa*) also restored the growth despite the extended lag periods and decreased growth rates compared to BG1-Rsb (Figure 4a). As expected, other ChlGs (*P. sitchensis*, *S. moellendorffii*, *P. patens*, *A. protothecoides*, *B. prasinos*, *C. crispus*, *C. merolae*, *Synechocystis*, and *C. tepidum*) did not support the photoheterotrophic growth of BG1 (Figure 4a,b). These results further corroborated the intracellular BChl *a*_P_ formation of BG1-carrying angiosperm *chlG*s (Figure 2).

Geranylgeranyl reductases (BchP and ChlP) reduce Chl *a*_GG_ to produce Chl *a*_P_. Alternatively, PPP is formed first from GGPP by BchP and ChlP and subsequently used as a substrate for ChlG to form Chl *a*_P_ [1,2] (Figure 1). The heterologous expression of *SychlG* with endogenous *bchP* in *R. sphaeroides* mutants grown under microoxic conditions, in which metabolic steps specific to BChl *a*_GG_ formation were interrupted, resulted in the formation of Chl *a*_P_ together with minor Chl *a* species possessing dihydroGG and tetrahydroGG groups [8]. Similarly, Chl *a*_P_ was formed predominantly by the various ChlGs examined in BZF1 grown in the dark in the presence of DMSO (75 mM) (Figure 3), which signifies the reduction in Chl *a*_GG_ to Chl *a*_P_ by BchP of *R. sphaeroides*. Minor Chl *a* was barely detectable in recombinant BZF1 cells (Figure 3), which may have been due to differences in the culture conditions.

### 3.2. NtChlG Exhibited the Highest Catalytic Efficiency for BChlide a among the Angiosperm ChlGs Examined

For functional comparison, kinetic analyses were performed using the angiosperm ChlGs (Table 2). RsBchG and SyChlG, which have the activities of BchG and ChlG alone, respectively, were included as controls. Both enzymes work in the ping-pong model using GGPP and PPP as the first substrate and BChlide *a* and/or Chlide *a* as the second substrate [2,26]. *K*_m_ and *k*_cat_ values were determined for BChlide *a* and Chlide *a* in the presence of GGPP and PPP, respectively. C-terminally His_6_-tagged BchG and ChlG were overexpressed in *E. coli,* and the cell lysates were used as enzyme sources. In order to calculate *k*_cat_, the amount of enzyme in the cell lysates was quantified via western blot analysis using an anti-His_6_-tag antibody. PpfC-His_6_ of similar size was used as the quantification standard (Figure S1).

NtChlG showed the highest catalytic efficiency for BchG activity among the angiosperm enzymes, which was two to three-fold higher than those of the other enzymes (Table 2). The kinetic properties of NtChlG2 for BChlide *a* and Chlide *a* (Table S4) were similar to those of NtChlG (Table 2). NtChlG had six to eight-fold higher *K*_m_ and two-fold lower *k*_cat_ for BChlide *a* compared to RsBchG, which resulted in approximately 12- to 18-fold lower catalytic efficiency (*k*_cat_/*K*_m_) (Table 2). Nevertheless, NtChlG supported the photoheterotrophic growth of BG1 (Figure 4a). Conversely, angiosperm ChlGs demonstrated catalytic efficiency for Chlide *a* comparable to that of SyChlG (Table 2).

The *k*_cat_/*K*_m_ values for BchG and ChlG activities were higher when GGPP was used instead of PPP. Consistently, lower *K*_m_ and higher *k*_cat_ were observed with GGPP when compared with the corresponding values with PPP. Thus, the geranylgeranylated pigment may be preferentially formed and subsequently converted to a phytylated pigment. The highest catalytic efficiency of NtChlG for BChlide *a* was also consistent with the shortest lag period and highest growth rate of BG1 expressing *NtchlG* (BG1-Ntc) among BG1 expressing other angiosperm *chlG* genes (Figure 4a).

Because angiosperm *chlG*s were expressed in *E. coli*, their BchG activities had to be confirmed directly in the plant. As a representative, *N. tabacum* plants were germinated and cultured in light for three weeks. The leaves were harvested, and their lysates were prepared and used as enzyme sources for analysis of native ChlG (Figure S2). The relative levels of NtChlG and NtChlG2 in the leaf lysates were not analyzed. However, the native leaf lysates exhibited intrinsic BchG activity. The apparent *K*_m_ values of the leaf lysates for BChlide *a* and Chlide *a* (Table S5) were not significantly different from those of NtChlG in the *E. coli* lysate (Table 2).

### 3.3. Angiosperm ChlGs Showed Resistance to Inhibition by Bacteriochlorin

**Figure 5:**
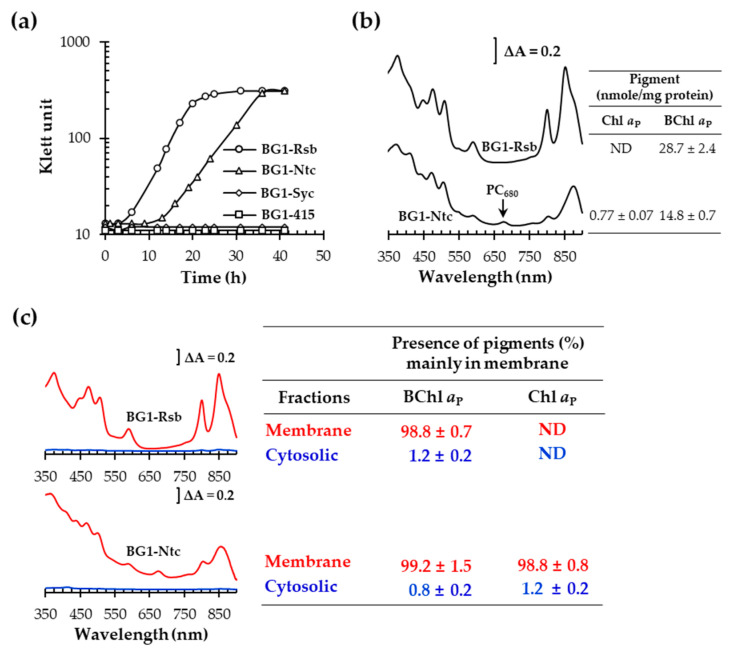
Effects of *NtchlG* expression on the photoheterotrophic growth of BG1. The photoheterotrophic growth BG1-Ntc (**a**) is shown with the absorption spectrum of the membrane fraction (**b**). BG1-Rsb, BG1-Syc, and BG1-415 were used as controls. Pigments were extracted from the membranes and quantified by HPLC; the obtained results are listed on the right side of the corresponding spectra (**b**). Membrane (red) and cytosolic fractions (blue) of photoheterotrophically growing BG1-Rsb and BG1-Ntc were separated and adjusted to the same volume as that (2 mL) used for resuspension of cells harvested for the analysis of absorption spectra (**c**). Pigments were extracted from the membrane, and cytosolic fractions and the relative levels of BChl *a*_P_ and Chl *a*_P_ were quantified by HPLC (**c**). BChl *a*_P_ and Chl *a*_P_ were detected at low levels in cytosolic fractions, which were ascribed to the contaminated membrane debris. ND, not detected.

To produce Chl *a*_P_ in *R. sphaeroides*, ChlG must be resistant to bacteriochlorin inhibition. The following clues suggested that angiosperm ChlGs would possess the resistance. First, AsChlG prenylated Chlide *a* in the presence of BChlide *a*, the amount of which was comparable to that of the reaction without BChlide *a* in a previous study [9]. This is unlike SyChlG, which is competitively inhibited by BChlide *a* [2]. Second, angiosperm ChlGs synthesized Chl *a*_P_ in BG1, which accumulates intracellular BChlide *a* (Figure 5b). Accordingly, ChlGs of angiosperm plants such as *A. thaliana*, *N. tabacum*, *A. sativa*, and *O. sativa* were examined for resistance to BChlide *a* inhibition to determine whether it is a common characteristic of the angiosperm enzymes. In order to select angiosperm ChlGs with the highest resistance to BChlide *a*, the inhibition constants (*K*_i_) for BChlide *a* were determined. Because ChlG is situated in the cell membrane, BChl *a*_GG_ and BChl *a*_P_ were also examined to determine whether they can act as inhibitors. Remarkably, SyChlG was competitively inhibited by BChlide *a* [2] (Figure S3g,j) and as well as by BChl *a*_GG_ and BChl *a*_P_ (Figure S3h,i,k,l). Similarly, RsBchG was competitively inhibited by Chlide *a* [2] (Figure S3a,d) and also by Chl *a*_GG_ and Chl *a*_P_ (collectively called chlorin) (Figure S3b,c,e,f). The *K*_i_ values of SyChlG for bacteriochlorin were similar to the corresponding constants of RsBchG for chlorin (Table 3). However, the *K*_i_ values of angiosperm ChlGs for bacteriochlorin were much larger than those of SyChlG (Table 3), indicating that the enzyme activities are highly resistant to bacteriochlorin. Among them, NtChlG had the highest *K*_i_ values for all bacteriochlorin inhibitors and was subsequently selected to produce Chl *a*_P_ under the biosynthetic pathway for BChl *a*_P_ of photoheterotrophically growing *R. sphaeroides* in the following experiments.

### 3.4. Heterologous Expression of NtchlG Resulted in the Formation of Free Chl a_P_ in R. sphaeroides while Generating ROS

The phenotype of BG1-Ntc (BG1 carrying *NtchlG*) was analyzed intensively with BG1-Rsb, BG1-Syc (BG1 carrying *SychlG*), and BG1-415 (BG1 carrying empty vector) as controls. The BG1-Ntc showed phototrophic growth with a 5- to 10-h lag period and a two-fold extended doubling time compared with that of BG1-Rsb (Figure 4a,c, and Figure 5a). As expected, SyChlG did not support the photoheterotrophic growth of BG1 (BG1-Syc) (Figure 4b and Figure 5a). A small peak was detected at 680 nm (PC_680_) in the membrane absorption spectrum of BG1-Ntc (Figure 5b), but no such peak was observed in the cytosolic fraction (Figure 5c). Chl *a*_P_ of BG1-Ntc amounted to 5% of BChl *a*_P_ (Figure 5b). As no PC_680_ was observed in the cytosolic fractions of BG1-Ntc (Figure 5c), the membrane fraction of BG1-Ntc was subjected to clear native polyacrylamide gel electrophoresis (CN-PAGE) [27] to analyze free pigments (Figure S4). Free Chl *a*_P_ migrates faster than protein-bound Chl *a*_P_ in CN-PAGE and can be easily detected in the lower part of the lanes via fluorescence under UV-A light. No free pigment was observed in BG1-Rsb, whereas free Chl *a*_P_ was detected in the BG1-Ntc. Similar bands were observed with BG1 carrying other angiosperm *chlGs* (Figure S4). Thus, the PC_680_ formation is ascribed to free Chl *a*_P_ accumulated in the membrane of cells grown under photoheterotrophic conditions. Formation of free Chl *a*_P_ at a much lower level (5%) than total BChl *a*_P_ further suggests that Chlide *a* is utilized to a small extent from the main metabolic stream toward BChlide *a* (Figure 5b).

In addition, WT-Ntc (WT carrying *NtchlG*) was analyzed with WT-Syc (WT carrying *SychlG*) and WT-415 (WT carrying empty vector) as controls. The growth of WT-Ntc was retarded compared to that of WT-415, whereas the growth of WT-Syc was not affected by the expression of the *chlG* gene (Figure S5a). PC_680_ was barely observed in the membrane spectrum of WT-Ntc (Figure S5b). Nonetheless, Chl *a*_P_ was still detected at only 2% of total BChl *a*_P_, which was lower than that of BG1-Ntc (5%). The absolute level of Chl *a*_P_ in WT-Ntc (0.65 ± 0.02 nmole/mg protein) was also lower than that in BG1-Ntc (0.77 ± 0.07), which was due to the altered metabolic flow to BChl *a*_P_ by the presence of ChlG in WT and BG1. Thus, we used BG1-Ntc to achieve higher Chl *a*_P_ formation in *R. sphaeroides*.

RsBchG was inhibited by chlorin to a similar degree as SyChlG was inhibited by bacteriochlorin (Table 3). However, BChl *a*_P_ levels in WT-Ntc were not different from that in the control cell WT-415 (Figure S5b). Therefore, BChl *a*_P_ formation by intrinsic RsBchG was unaffected by the heterologous expression of *NtchlG* in *R. sphaeroides*, which was due to the low level (2%) of Chl *a*_P_ formation compared with BChl *a*_P_ (Figure S5b).

**Figure 6:**
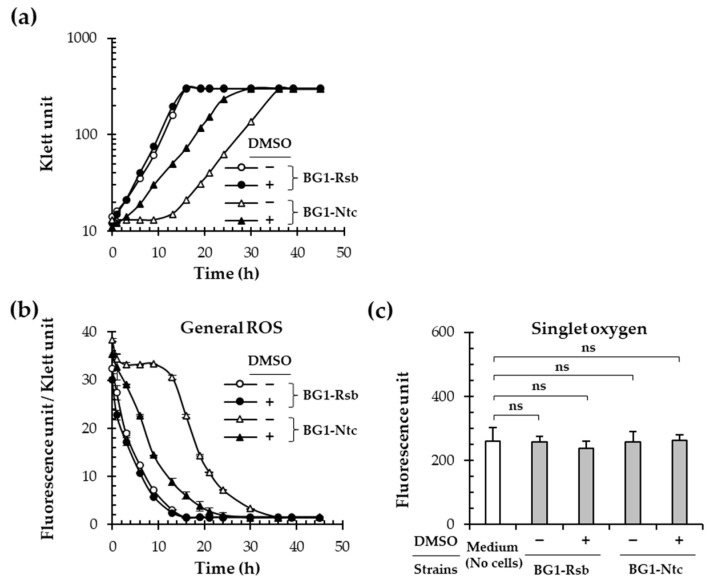
The photoheterotrophic growth of BG1-Ntc (**a**) was examined in the presence (closed symbol, +) or absence (open symbol, −) of 2 mM DMSO. BG1-Rsb was used as the control. General ROS levels were determined during the growth period using H_2_DCFDA (**b**). Singlet oxygen levels were determined using SOSG 1 h after the onset of photoheterotrophic growth (**c**). Significances were assessed using the Student’s *t*-test: ns, *p* > 0.05.

Next, we aimed to elucidate why the retarded growth rate and extended lag period were observed in BG1-Ntc (Figure 1 and Figure 5a). Since aerobically grown seed cells were routinely inoculated into a fresh medium for the main culture at a 5% volume ratio, oxygen could be introduced into the photoheterotrophic culture of BG1-Ntc. Because free Chl *a*_P_ was present in the membranes of photoheterotrophically growing BG1-Ntc (Figure 5b and Figure S4), the general ROS levels were examined during photoheterotrophic growth. The general ROS levels of BG1-Ntc were higher than those of BG1-Rsb during the lag period and exponential growth (Figure 6b). Since DMSO is known to scavenge hydroxyl radicals [13], 2 mM DMSO was added at the onset of the photoheterotrophic culture. While the growth of the control cells (BG1-Rsb) was unaffected by the addition of 2 mM DMSO (Figure 6a), the general ROS level was decreased to a larger extent by 2 mM DMSO in BG1-Ntc (Figure 6b), resulting in the disappearance of the growth lag (Figure 6a). However, the cell growth rate remained unaffected by treatment with DMSO. Singlet oxygen was measured using SOSG, and no difference was observed between the cells examined (Figure 6c). Thus, the prolonged lag of BG1-Ntc resulted due to the elevated levels of general ROS; however, the reason for the slow growth rate during the exponential phase remains unclear.

## 4. Discussion

Chl *a* production in *R. sphaeroides* would be the first step in the construction of Chl *a*_P_-containing PS complexes. Recently, water-soluble Chl-binding protein (WSCP) of *Brassica oleracea* was produced in *R. sphaeroides* mutants that accumulated Chlide *a* [8], and the inhibition of SyChlG by BChlide *a* and BChl *a*_P_ was prevented by blocking the metabolic steps specific to BChlide *a* formation. Consequently, the resulting mutant lacked a BChl *a*_P_-containing PS complex [8]. Interestingly, heterologous expression of *Prochlorococcus marinus* (a cyanobacterium) *chlG* (*PmchlG*) in *R. sphaeroides* WT resulted in Chl *a* accumulation [28]. However, the initial attempt to produce Chl *a*_P_ using PmChlG failed in both the WT and BG1 strains (Figure S6). Chl *a*_P_ expression was observed in BZF1 alone, which did not produce BChlide *a* (Figure S6b). The differences between previous reports [28] and our study remain to be determined.

The results demonstrate that angiosperm ChlGs possess BchG activity. The physiological relevance of this activity in plants is unknown. Evolutionary analysis of ChlGs and BchGs using the maximum likelihood method with the Le-Gascuel model [29] revealed that angiosperm ChlGs constitute a subclade distinct from the other ChlGs examined (Figure S7). Moreover, resistance to inhibition by bacteriochlorin (BChlide *a*, BChl *a*_GG_, and BChl *a*_P_) is specific to angiosperm ChlGs, and this property is distinct from other ChlGs, including SyChlG. The two properties (BchG activity and resistance of ChlG activity to bacteriochlorin inhibition) of angiosperm ChlGs are likely to be correlated through the interaction of the substrate and inhibitor at the active site of the enzyme. Nevertheless, the exact biochemical correlation requires structural elucidation of ChlG and BchG.

Chlide *a* (C3-vinyl, C7-methyl, C7–8 double bond) and BChlide *a* (C3-acetyl, C7-methyl, C7–8 single bond) have structural differences in the functional group at C3 (ring A) and the reduction state between C7 and C8 (ring B) (Figure 1). The structural differences between Chlide *a* and BChlide *a* may be distinguished by the active sites of ChlG and BchG. AsChlG activity was examined using Chlide *a*, BChlide *a*, Chlide *b* (C3-vinyl, C7-aldehyde, C7–8 double bonds), and acetyl-Chlide *a* (C3-acetyl, C7-methyl, C7–8 double bond) [9]. The activity of AsChlG for Chlide *b* and acetyl-Chlide *a* was comparable to that of Chlide *a*, whereas that of BChlide *a* was reduced [9]. This result is corroborated by the lower specificity constant of AsChlG for BChlide *a* compared to that for Chlide *a*, and similar results were obtained for other angiosperms ChlGs (Table 2). Furthermore, AsChlG was able to utilize the Chlide *a*-derivative as a substrate, in which C7 was exchanged to hold a bulky phenylamino group. The resulting activity was modestly lower than that observed for Chlide *a* [30]. Thus, AsChlG activity was largely unaffected when the Chlide substrate was altered at C3 and C7, but the state of reduction between C7–8 was critical for the activity of AsChlG.

AsChlG has been shown to have higher activity in the presence of GGPP than in the presence of PPP [1]. Similarly, RsBchG and SyChlG showed lower *K*_m_ values for BChlide *a* and Chlide *a*, respectively, in the presence of GGPP [2], and similar results were obtained for angiosperm ChlGs (Table 2). Thus, GGPP is preferred relative to PPP by BchG and ChlGs examined in this study.

Remarkably, RsBchG and SyChlG were inhibited by Chl *a* and BChl *a*, respectively (Table 3). When the C_20_ moiety of the inhibitor (Chl *a*/BChl *a*) was the same as that of the first substrate (GGPP and PPP), the inhibition constant *K*_i_ was higher than that observed when they were different. Although a detailed interpretation of the results requires structural elucidation of both ChlG and BchG, the results may reflect the interaction between the prenylated active site and the prenyl group of the inhibitor (Chl *a*/BChl *a*).

The photoheterotrophic growth of *R. sphaeroides* was affected when *NtchlG* was expressed. As growth retardation was not observed with *SychlG* (Figure S5), Chl *a*_P_ accumulation was thought to affect cell growth. Free Chl *a*_P_ generated ROS and DMSO (2 mM) was used to prevent oxidative damage. However, DMSO failed to restore the growth rate to that observed in control. One possible cause is the inhibition of BChl *a*_P_ incorporation into the PS complex by Chl *a*_P_. The reaction center (RC) complex of *R. sphaeroides* has four BChl *a*_P_ and two bacteriopheophytin *a*_P_ (Bpheo *a*_P_, Mg^2+^-free form of BChl *a*_P_) molecules. When pheophytin *a*_P_ (Pheo *a*_P_, Mg^2+^-free form of Chl *a*_P_) is provided to an isolated RC complex, the Bpheo *a*_P_ of the RC complex can be easily replaced by Pheo *a*_P_ in the presence of a mild detergent, such as lauryldimethylamine oxide, with heating at 42 °C [31,32,33,34,35]. The modified RC complex containing Pheo *a*_P_ illustrated the electron transfer kinetics with a decreased quantum yield for both triplet special pair formation and quinone reduction compared with the native RC [34,35]. BChl *a*_P_ of light-harvesting complex 2 (LH2) complex of *R. sphaeroides* can also be exchanged for Chl *a*_P_, which was proposed to exhibit carotenoid-to-BChl energy transfer that was different from the native complex [36,37]. Thus, Chl *a*_P_ produced by NtChlG can affect the binding of BPheo *a*_P_ and BChl *a*_P_ to the RC and LH2 complexes, respectively, resulting in unexpected detrimental effects on PS growth.

## 5. Conclusions

In this study, angiosperm ChlGs were kinetically characterized and found to exhibit ChlG activities that are resistant to bacteriochlorin inhibitions. Among the angiosperms ChlGs studied, *N. tabacum chlG* demonstrated the best activity, and its expression produced Chl *a*_P_ in *R. sphaeroides* under photoheterotrophic conditions in which BChl *a*_P_ coexisted. The results of this study will be useful for building functional Chl *a*_P_-containing LHC in *R. sphaeroides*, which is currently underway.

## Figures and Tables

**Table 1:** BchG and ChlG activities of enzymes annotated as BchG and ChlG from various PS organisms.

Classification			Species	Enzyme	BchG Activity	ChlG Activity
Vascular plant	Angiosperm	Eudicot	*Arabidopsis thaliana*	ChlG	+	+
			*Nicotiana tabacum*	ChlG	+	+
				ChlG2	+	+
		Monocot	*Avena sativa*	ChlG	+ ^a^	+
			*Oryza sativa*	ChlG	+	+
	Gymnosperm		*Picea sitchensis*	ChlG	−	+
	Lycophyte		*Selaginella moellendorffii*	ChlG	−	+
Bryophyte	Moss		*Physcomitrella patens*	ChlG	−	+
Algae	Chlorophyte (green algae)		*Auxenochlorella protothecoides*	ChlG	−	+
			*Bathycoccus prasinos*	ChlG	−	+
	Rhodophyte (red algae)		*Chondrus crispus*	ChlG	−	+
			*Cyanidioschyzon merolae*	ChlG	−	+
PS bacteria	Cyanobacteria		*Synechocystis* sp. PCC6803	ChlG	−	+
	Purple nonsulfur bacteria		*Rhodobacter sphaeroides*	BchG	+	−
	Purple sulfur bacteria		*Allochromatium vinosum*	BchG	+	−
	Green nonsulfur bacteria		*Chloroflexus aurantiacus*	BchG	+	−
	Green sulfur bacteria		*Chlorobaculum tepidum*	BchG	+	−
				ChlG	−	+
	Aerobic anoxygenic PS bacteria		*Roseobacter denitrificans*	BchG	+	−

^a^ BchG activity of *A. sativa* ChlG was reported previously [9].

**Table 2:** Kinetic parameters of BchG and ChlGs for BChlide *a* and Chlide *a*.

Enzyme ^a^	First Substrate	Second Substrate	*K*_m_ (μM)	*k*_cat_ (min^−1^)	*k*_cat_/*K*_m_ (mM^−1^ min^−1^)
BchG (*R. sphaeroides*)	GGPP	BChlide *a*	23 ± 2	4.6 ± 0.2	201 ± 7
	PPP		37 ± 2	3.4 ± 0.5	92 ± 11
ChlG (*Synechocystis*)	GGPP	Chlide *a*	14 ± 2	17.1 ± 0.9	1193 ± 59
	PPP		47 ± 1	15.8 ± 0.3	337 ± 6
ChlG (*A. thaliana*)	GGPP	BChlide *a*	549 ± 34	2.2 ± 0.1	4 ± 1
	PPP		611 ± 33	1.7 ± 0.3	3 ± 1
	GGPP	Chlide *a*	10 ± 1	10.6 ± 0.3	1084 ± 32
	PPP		22 ± 1	9.0 ± 0.1	412 ± 4
ChlG (*N. tabacum*)	GGPP	BChlide *a*	180 ± 19	2.0 ± 0.1	11 ± 1
	PPP		211 ± 30	1.6 ± 0.2	8 ± 1
	GGPP	Chlide *a*	10 ± 2	9.3 ± 0.7	932 ± 68
	PPP		24 ± 1	7.8 ± 0.1	330 ± 2
ChlG (*A. sativa*)	GGPP	BChlide *a*	398 ± 17	2.2 ± 0.1	6 ± 1
	PPP		483 ± 27	1.7 ± 0.3	4 ± 1
	GGPP	Chlide *a*	12 ± 1	14.7 ± 0.5	1213 ± 39
	PPP		29 ± 3	11.6 ± 0.1	398 ± 4
ChlG (*O. sativa*)	GGPP	BChlide *a*	413 ± 17	2.4 ± 0.1	6 ± 1
	PPP		621 ± 10	1.8 ± 0.3	3 ± 1
	GGPP	Chlide *a*	9 ± 1	12.5 ± 0.3	1370 ± 34
	PPP		23 ± 1	10.5 ± 0.1	457 ± 5

^a^ *E. coli* lysates containing ChlG were used as enzyme sources.

**Table 3:** Inhibition constants of BchG and ChlGs for bacteriochlorin and chlorin.

Enzyme ^a^	First Substrate	Second Substrate	*K*_i_ (μM) ^b^					
			BChlide *a*	Chlide *a*	BChl *a*_GG_	Chl *a*_GG_	BChl *a*_P_	Chl *a*_P_
BchG (*R. sphaeroides*)	GGPP	BChlide *a*	NA ^c^	**35 ± 3 ^d^**	797 ± 96	**75 ± 6 ^d^**	673 ± 46	**65 ± 4 ^d^**
	PPP		NA	**73 ± 5 ^d^**	623 ± 25	**62 ± 5 ^d^**	676 ± 17	**100 ± 6 ^d^**
ChlG (*Synechocystis*)	GGPP	Chlide *a*	**30 ± 1 ^d^**	NA	**63 ± 4 ^d^**	688 ± 20	**40 ± 3 ^d^**	583 ± 35
	PPP		**97 ± 4 ^d^**	NA	**43 ± 3 ^d^**	596 ± 47	**105 ± 4 ^d^**	750 ± 67
ChlG (*A. thaliana*)	GGPP	Chlide *a*	412 ± 47	NA	318 ± 41	695 ± 85	248 ± 37	596 ± 41
	PPP		587 ± 10	NA	201 ± 16	631 ± 66	265 ± 9	806 ± 20
ChlG (*N. tabacum*)	GGPP	Chlide *a*	502 ± 57	NA	395 ± 47	665 ± 59	360 ± 36	612 ± 28
	PPP		681 ± 26	NA	348 ± 41	617 ± 35	484 ± 42	796 ± 17
ChlG (*A. sativa*)	GGPP	Chlide *a*	379 ± 37	NA	306 ± 31	808 ± 53	276 ± 24	785 ± 59
	PPP		490 ± 36	NA	242 ± 19	661 ± 82	308 ± 21	723 ± 45
ChlG (*O. sativa*)	GGPP	Chlide *a*	375 ± 54	NA	319 ± 39	705 ± 22	265 ± 31	646 ± 51
	PPP		561 ± 47	NA	271 ± 29	595 ± 47	341 ± 23	703 ± 20

^a^ *E. coli* lysates containing ChlGs were used as enzyme sources. ^b^ Competitive inhibition constants. ^c^ Not applicable. ^d^ Relatively strong inhibitions (*K*_i_ < 100).

## Data Availability

Not applicable.

## Supplementary Materials

The following supporting information can be downloaded at: https://www.mdpi.com/article/10.3390/biology12040573/s1. Table S1: Bacterial strains; Table S2: Primers; Table S3: Plasmids; Table S4: Kinetic parameters of NtChlG2; Figure S1: Quantification of BchG and ChlG in *E. coli* lysate; Figure S2: ChlG and BchG activities in leaf lysates of *N. tabacum*; Table S5: *K*_m_ of native ChlG from leaf lysates of *N. tabacum* for BChlide *a* and Chlide *a*; Figure S3: Inhibition plots of RsBchG and SyChlG for chlorin and bacteriochlorin, respectively; Figure S4: Detection of free Chl *a*_P_ in the membranes of BG1 expressing angiosperm *chlG*s; Figure S5: Effect of *NtchlG* expression on the photoheterotrophic growth of WT; Figure S6: Chl *a*_P_ formation by *Prochlorococcus marinus* ChlG in *R. sphaeroides*; Figure S7: Evolutionary analysis of BchGs and ChlGs; Amino acid sequence of BchG and ChlG; Construction of plasmids; Clear-native PAGE; Kinetic analysis of BchG and ChlG activities in leaf extract of *N. tabacum*. References [2,8,11,16,17,27,29,38,39,40,41,42] are cited in the supplementary materials.
