# Supplementary material for: Chlorophyll a Synthesis in Rhodobacter sphaeroides by Chlorophyll Synthase of Nicotiana tabacum
Source: Biology (Basel). 2023 Apr 10;12(4):573. doi: 10.3390/biology12040573 (PMC10136183; doi:10.3390/biology12040573)
Supplement: Supplementary file 1 [file biology-12-00573-s001.zip › biology-2340735-supplementary.pdf]

Table S1. Bacterial strains.

Strains	Relevant characteristics	Source or reference
R. sphaeroides		
R. sphaeroides 2.4.1	Type strain	[1]
BG1	Sm^r/Sp^r; Δ*bchG* mutant; gene interruption in *bchG* by Sm^r/Sp^r cassette	[2]
BF	Δ*bchF* mutant; internal deletion in *bchF* by pLOtp-ΔbchF [3]	This study
BZF1	Km^r; Δ*bchZ* Δ*bchF* mutant; internal deletion in *bchF* and interruption in *bchZ* by Km^r cassette	[3]
BP	Δ*bchP* mutant; internal deletion	This study
BFP	Δ*bchF* Δ*bchP* mutant; internal deletion in *bchF* and *bchP*	This study
BZFP	Km^r; Δ*bchZ* Δ*bchF* Δ*bchP* mutant; internal deletion in *bchF* and *bchP*, interruption in *bchZ* by Km^r cassette	This study
BZFP-WSCP-Ntc	Km^r, Tc^r; BZFP + pRK-WSCP-Ntc	This study
BG1-415	Sm^r/Sp^r, Tc^r; BG1 + pRK415	This study
BG1-Atc	Sm^r/Sp^r, Tc^r; BG1 + pRK-Atc	This study
BG1-Ntc	Sm^r/Sp^r, Tc^r; BG1 + pRK-Ntc	This study
BG1-Asc	Sm^r/Sp^r, Tc^r; BG1 + pRK-Asc	This study
BG1-Osc	Sm^r/Sp^r, Tc^r; BG1 + pRK-Osc	This study
BG1-Rsb	Sm^r/Sp^r, Tc^r; BG1 + pRK-Rsb	This study
BG1-Syc	Sm^r/Sp^r, Tc^r; BG1 + pRK-Syc	This study
BG1-Psc	Sm^r/Sp^r, Tc^r; BG1 + pRK-Psc	This study
BG1-Smc	Sm^r/Sp^r, Tc^r; BG1 + pRK-Smc	This study
BG1-Ppc	Sm^r/Sp^r, Tc^r; BG1 + pRK-Ppc	This study
BG1-Apc	Sm^r/Sp^r, Tc^r; BG1 + pRK-Apc	This study
BG1-Bpc	Sm^r/Sp^r, Tc^r; BG1 + pRK-Bpc	This study
BG1-Ccc	Sm^r/Sp^r, Tc^r; BG1 + pRK-Ccc	This study
BG1-Cmc	Sm^r/Sp^r, Tc^r; BG1 + pRK-Cmc	This study
BG1-Avb	Sm^r/Sp^r, Tc^r; BG1 + pRK-Avb	This study
BG1-Cab	Sm^r/Sp^r, Tc^r; BG1 + pRK-Cab	This study
BG1-Ctb	Sm^r/Sp^r, Tc^r; BG1 + pRK-Ctb	This study
BG1-Ctc	Sm^r/Sp^r, Tc^r; BG1 + pRK-Ctc	This study
BG1-Rdb	Sm^r/Sp^r, Tc^r; BG1 + pRK-Rdb	This study
BG1-Pmc	Sm^r/Sp^r, Tc^r; BG1 + pRK-Pmc	This study
WT-415	Tc^r; WT + pRK415	This study
WT-Syc	Tc^r; WT + pRK-Syc	This study
WT-Ntc	Tc^r; WT + pRK-Ntc	This study
WT-Pmc	Tc^r; WT + pRK-Pmc	This study
BZF1-415	Km^r, Tc^r; BZF1 + pRK415	This study
BZF1-Atc	Km^r, Tc^r; BZF1 + pRK-Atc	This study
BZF1-Ntc	Km^r, Tc^r; BZF1 + pRK-Ntc	This study
BZF1-Asc	Km^r, Tc^r; BZF1 + pRK-Asc	This study
BZF1-Osc	Km^r, Tc^r; BZF1 + pRK-Osc	This study
BZF1-Rsb	Km^r, Tc^r; BZF1 + pRK-Rsb	This study
BZF1-Syc	Km^r, Tc^r; BZF1 + pRK-Syc	This study
BZF1-Psc	Km^r, Tc^r; BZF1 + pRK-Psc	This study
BZF1-Smc	Km^r, Tc^r; BZF1 + pRK-Smc	This study
BZF1-Ppc	Km^r, Tc^r; BZF1 + pRK-Ppc	This study
BZF1-Apc	Km^r, Tc^r; BZF1 + pRK-Apc	This study
BZF1-Bpc	Km^r, Tc^r; BZF1 + pRK-Bpc	This study
BZF1-Ccc	Km^r, Tc^r; BZF1 + pRK-Ccc	This study
BZF1-Cmc	Km^r, Tc^r; BZF1 + pRK-Cmc	This study
BZF1-Avb	Km^r, Tc^r; BZF1 + pRK-Avb	This study
BZF1-Cab	Km^r, Tc^r; BZF1 + pRK-Cab	This study
BZF1-Ctb	Km^r, Tc^r; BZF1 + pRK-Ctb	This study
BZF1-Ctc	Km^r, Tc^r; BZF1 + pRK-Ctc	This study
BZF1-Rdb	Km^r, Tc^r; BZF1 + pRK-Rdb	This study
BZF1-Pmc	Km^r, Tc^r; BZF1 + pRK-Pmc	This study
E. coli		
DH5α *phe*	*sup*E44 Δ*lacU*169 (Φ80 *lacZ* ΔM15) *hsd*R17 *recA1 endA1 gyrA96 thi*-1 *rel*A1 *phe*::Tn*10d*Cm	[4]
S17-1	C600::RP-4 2-(Tc::Mu)(Km::Tn7) *thi pro hsdR hsdM+ recA*	[5]
BL21 (DE3)	*E. coli* B F− *dcm ompT hsdS*(rB− mB−) gal λ(DE3)	Stratagene

Table S2. Primers.

Primers	Nucleotide sequence (from 5′ end to 3′ end)	Note
P1F	GCATGCCGCTGATCGGCCAGATGC	*Sph* Ⅰ is underlined.
P1R	GAATTCCACGATCTGGCTGTCGGG	*Eco*R Ⅰ is underlined.
P2F	GAATTCGTTCTCCGCTCGATGCAG	*Eco*R Ⅰ is underlined.
P2R	TCTAGAACGCGGTCGGCGAAGACG	*Xba* Ⅰ is underlined.
P3F	GGTCTCGAATGGCGGCGATAGAGGAC	*Bsa*I is underlined.
P3R	GGTCTCAGCGCTGACGAAGATACCAGAAGCTTC	*Bsa*I is underlined.
P4F	GGTACCCACGCCCTGAATGTGGGC	*Kpn*I is underlined.
P4R	TCTAGAGTCGGCCTGCACGCGGGC	*Xba*I is underlined.
P5F	GGTACCCACGCCCTGAATGTGGGC	*Bam*HI is underlined.
P5R	AAAGAATTCTCACGGCAGCACCTCCAGCCC	*Eco*RI is underlined.
P6F	AAAGGATCCGAACTTTCCTCTCAATTC	*Bam*HI is underlined.
P6R	AAAGAATTCTCAAATCCCCGCATGGCC	*Eco*RI is underlined.
P7F	TTTCATATGAGTGTCAATCTATCC	*Nde*I is underlined.
P7R	TTTAAGCTTCGGCAGCACCTCCAGCCC	*Hin*dIII is underlined.
P8F	TTTCATATGTCTGACACACAAAATACC	*Nde*I is underlined.
P8R	TTTAAGCTTAATCCCCGCATGGCCTAG	*Hin*dIII is underlined.
P9F	CATATGACCAGCATTCTGAAC	*Nde*I is underlined.
P9R	AAGCTTGTGTTGCGATGCTAATGC	*Hin*dIII is underlined.
P10F	CATATGGCGAGCCTGCTGAAC	*Nde*I is underlined.
P10R	AAGCTTATGGGATGTTGCCAGCGC	*Hin*dIII is underlined.
P11F	CATATGGCGACCAGCCATCCG	*Nde*I is underlined.
P11R	AAGCTTATGGCTAGTCGCCAGTGC	*Hin*dIII is underlined.
P12F	CATATGGCGACCAGCCATCTG	*Nde*I is underlined.
P12R	AAGCTTATGACTGGTAGCTAATGC	*Hin*dIII is underlined.

Table S3. Plasmids.

Plasmids	Relevant characteristics	Source of reference
pLO1	Km^r; *sacB*$^+$, RP4 *oriT*, ColE1 *ori*; suicide vector for mutant construction	[6]
pRK415	Tc^r; *ori* IncP Mob RP4 *lacZα*; expression vector for *R. sphaeroides*	[7]
pASK-IBA3plus	Ap^r; inducible expression vector for strep-tagged protein in *E. coli*	IBA Life sciences
pET29a	Km^r; inducible expression vector for His$_6$-tagged protein in *E. coli*	Novagen
pLO-bchP	pLO1 + 1,046-bp *Sph*I/*Xba*I fragment containing internally deleted *bchP* gene	This study
pChlase	pASK-IBA3plus + 972-bp *Bsa*I fragment containing *A. thaliana* chlorophyllase gene	This study
pRK-WSCP-Ntc	pRK415 + 2,337-bp *Kpn*I/*Eco*RI fragment containing WSCP gene and *N. tabacum chlG*	This study
pRK-Rsb	pRK415 + 1,042-bp *Bam*HI/*Eco*RI fragment containing *R. sphaeroides bchG*	This study
pRK-Syc	pRK415 + 1,118-bp *Bam*HI/*Eco*RI fragment containing *Synechocystis chlG*	This study
pRK-Atc	pRK415 + 1,184-bp *Xba*I/*Eco*RI fragment containing *A. thaliana chlG*	This study
pRK-Ntc	pRK415 + 1,142-bp *Xba*I/*Eco*RI fragment containing *N. tabacum chlG*	This study
pRK-Asc	pRK415 + 1,157-bp *Xba*I/*Eco*RI fragment containing *A. sativa chlG*	This study
pRK-Osc	pRK415 + 1,151-bp *Xba*I/*Eco*RI fragment containing *O. sativa chlG*	This study
pRK-Psc	pRK415 + 1,187-bp *Xba*I/*Eco*RI fragment containing *P. sitchensis chlG*	This study
pRK-Smc	pRK415 + 1,193-bp *Xba*I/*Eco*RI fragment containing *S. moellendorffii chlG*	This study
pRK-Ppc	pRK415 + 1,811-bp *Xba*I/*Eco*RI fragment containing *P. patens chlG*	This study
pRK-Apc	pRK415 + 1,232-bp *Xba*I/*Eco*RI fragment containing *A. protothecoides chlG*	This study
pRK-Bpc	pRK415 + 1,295-bp *Xba*I/*Eco*RI fragment containing *B. prasinos chlG*	This study
pRK-Ccc	pRK415 + 1,049-bp *Xba*I/*Eco*RI fragment containing *C. crispus chlG*	This study
pRK-Cmc	pRK415 + 1,232-bp *Xba*I/*Eco*RI fragment containing *C. m*erola*e chlG*	This study
pRK-Avb	pRK415 + 935-bp *Xba*I/*Eco*RI fragment containing *A. vinosum bchG*	This study
pRK-Cab	pRK415 + 953-bp *Xba*I/*Eco*RI fragment containing *C. aurantiacus bchG*	This study
pRK-Ctb	pRK415 + 1,013-bp *Xba*I/*Eco*RI fragment containing *C. tepidum bchG*	This study
pRK-Ctc	pRK415 + 1,124-bp *Xba*I/*Eco*RI fragment containing *C. tepidum chlG*	This study
pRK-Rdb	pRK415 + 920-bp *Xba*I/*Eco*RI fragment containing *R. denitrificans bchG*	This study
pRK-Pmc	pRK415 + 971-bp *Xba*I/*Eco*RI fragment containing *P. marinus chlG*	This study
pET-Rsb	pET29a + 906-bp *Nde*I/*Hin*dIII fragment containing *R. sphaeroides bchG*	This study
pET-Syc	pET29a + 972-bp *Nde*I/*Hin*dIII fragment containing *Synechocystis* sp. PCC6803 *chlG*	This study
pET-Atc	pET29a + 1,161-bp *Nde*I/*Hin*dIII fragment containing *A. thaliana chlG*	This study
pET-Ntc	pET29a + 1,119-bp *Nde*I/*Hin*dIII fragment containing *N. tabacum chlG*	This study
pET-Ntc2	pET29a + 1,119-bp *Nde*I/*Hin*dIII fragment containing *N. tabacum chlG2*	This study
pET-Asc	pET29a + 1,134-bp *Nde*I/*Hin*dIII fragment containing *A. sativa chlG*	This study
pET-Osc	pET29a + 1,128-bp *Nde*I/*Hin*dIII fragment containing *O. sativa chlG*	This study

Table S4. Kinetic parameters of NtChlG2.

First substrate[a]	Second substrate[a]	K_m (μM)	k_{cat} (min^{-1})	k_{cat} / K_m (mM^{-1} min^{-1})
**PPP**	**BChlide *a***	196 ± 31	1.9 ± 0.3	10 ± 2
	**Chlide *a***	23 ± 1	8.7 ± 0.1	378 ± 4

[a]*E. coli* lysate containing NtChlG2 was used as an enzyme source.

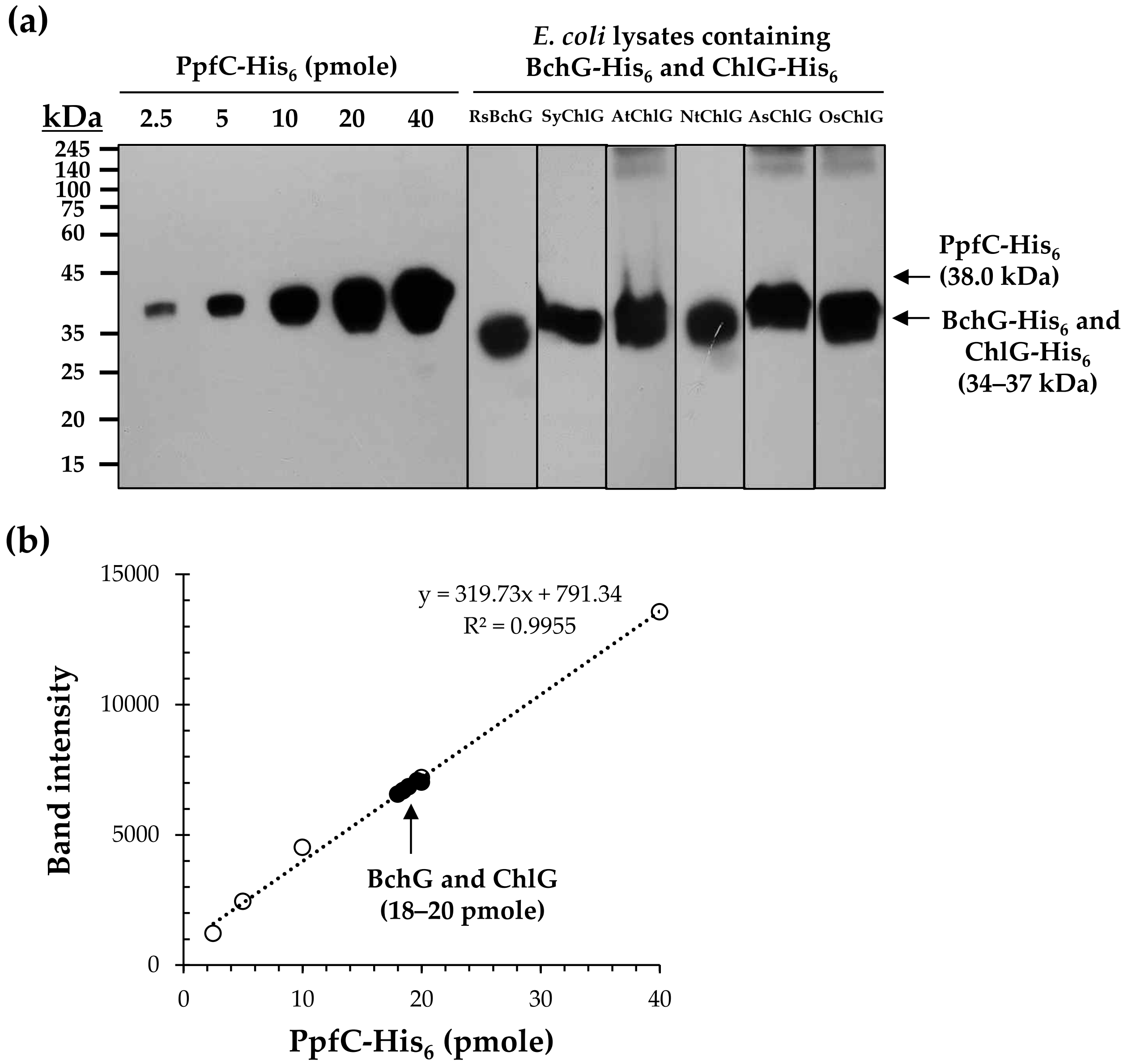

Figure S1. Quantification of BchG and ChlG in *E. coli* lysate.

BchG and ChlGs were fused to His_6-tag at C-termini. BchG-His_6 and ChlG-His_6 in lysates of *E. coli* BL21 (DE3) were quantified by western immunoblot analysis using anti-His_6-tag antibody. Cell lysates were loaded on SDS-polyacrylamide gel in parallel with varying amounts of PpfC-His_6 (38.0 kDa) as a quantification standard (a). The intensity of bands on expected size were measured by densitometer, and used to construct standard curve (b). The calculated amount of RsBchG, SyChlG, AtChlG, NtChlG, AsChlG, and OsChlG in cell lysates (0.2 mg protein) were 18.0, 19.6, 18.5, 18.4, 18.9, and 20.0 pmole, respectively.

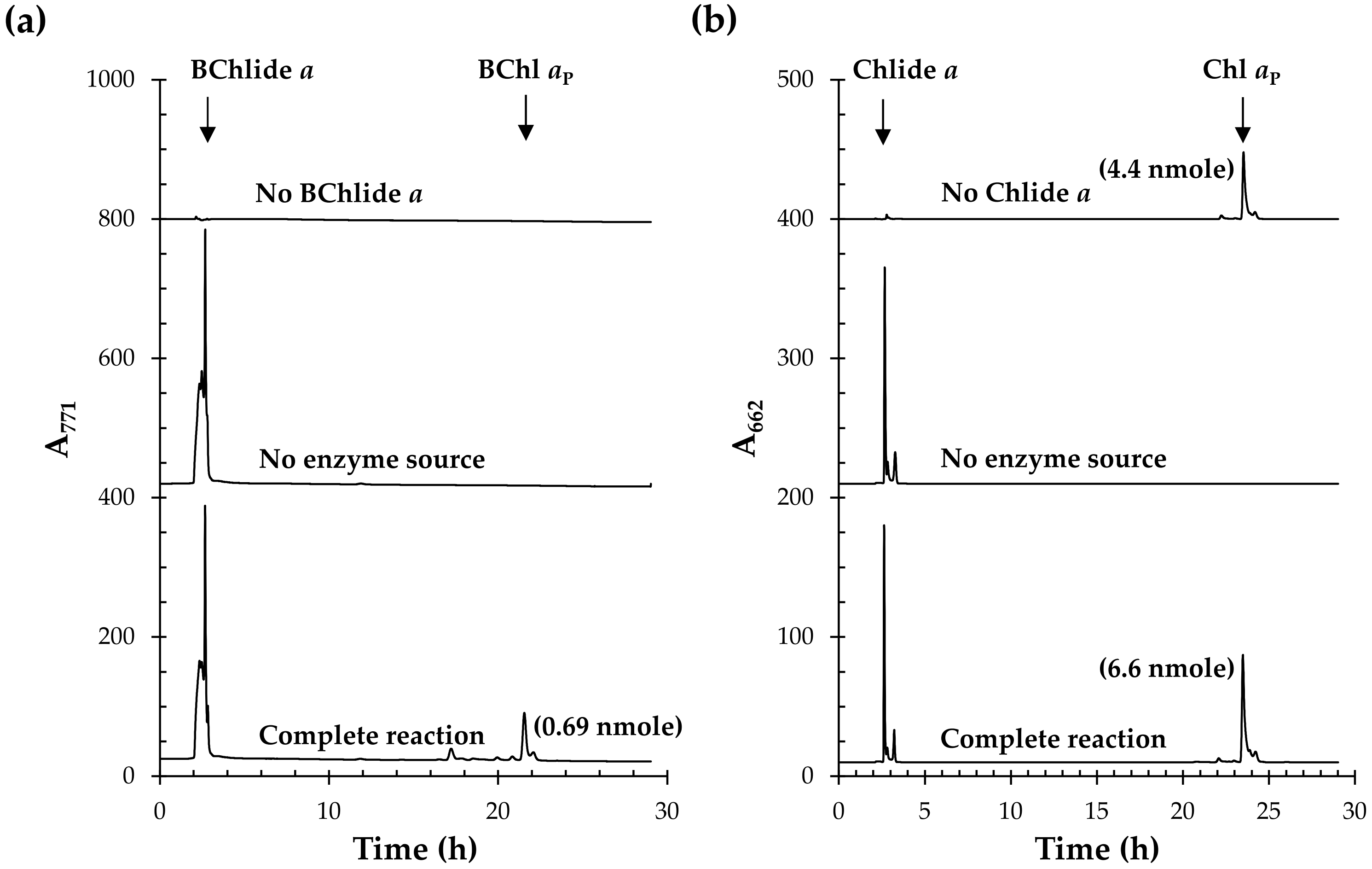

Figure S2. ChlG and BchG activities in leaf lysates of *N. tabacum*.

Light-incubated *N. tabacum* leaves (3-week-old) were lysed by sonication. Leaf lysates (1 mg protein) were used as enzyme sources for BchG (a) and ChlG reaction (b). PPP was used as the first substrate. BchG activity was confirmed by the formation of BChl a_P peak that is detected only in the presence of the second substrate BChlide *a* and leaf lysates (complete reaction). "No substrate" (no BChlide *a*) (left top) and "No enzyme source" (no leaf lysates) (left middle) were included as controls. ChlG assay was performed similarly. Chl a_P was detected without addition of Chlide *a* exogenously, which was ascribed to the residual Chl a_P in the enzyme source (right top). Elevated formation of Chl a_P was observed when Chlide *a* was exogenously added to the reaction mixture, as expected (right bottom).

Table S5. K_m of native ChlG from leaf lysates of *N. tabacum* for BChlide *a* and Chlide *a*.

Enzyme	First substrate	Second substrate	K_m (μM)
**Leaf lysate**[a]	**PPP**	**BChlide *a***	257 ± 31
		**Chlide *a***	14 ± 1

[a]Light-incubated leaves of *N. tabacum* were used.

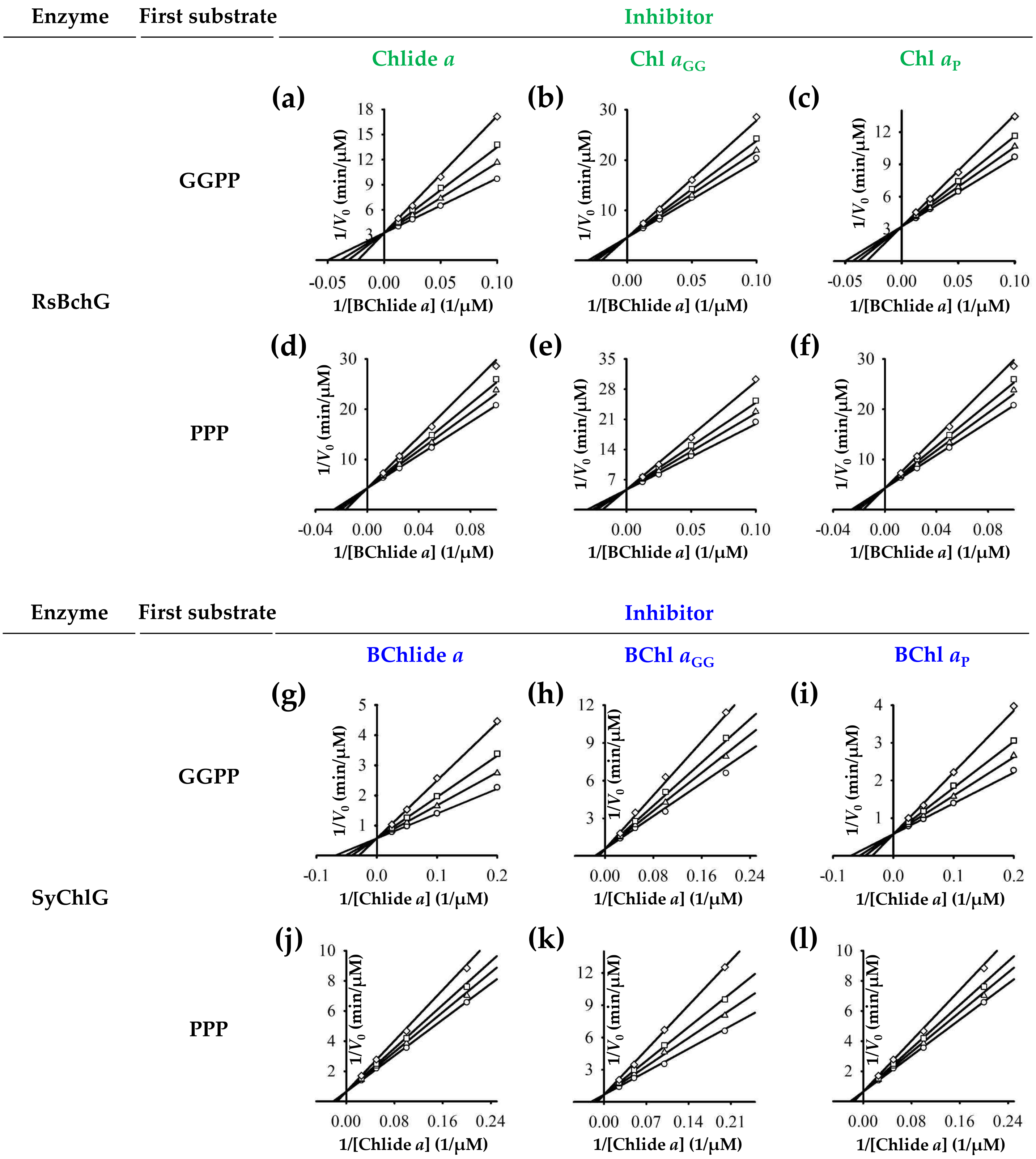

Figure S3. Inhibition plots of RsBchG and SyChlG for chlorin and bacteriochlorin, respectively.

Inhibition of RsBchG (a–f) by Chlide *a* (a and d), Chl a_{GG} (b and e) and Chl a_{P} (c and f), and that of SyChlG (g–l) by BChlide *a* (g and j), BChl a_{GG} (h and k) and BChl a_{P} (i and l) with GGPP (50 μM) (a–c and g–i) or PPP (50 μM) (d–f and j–l) were illustrated by double reciprocal plots of the second substrate (BChlide *a* or Chlide *a*) concentrations and initial velocities (V_0). Inhibitors were used at 0 (circle), 10 (triangle), 20 (square) and 40 (diamond) μM. All datasets were fitted to competitive inhibition model by nonlinear regression. Computed inhibition constants were listed in Table 3.

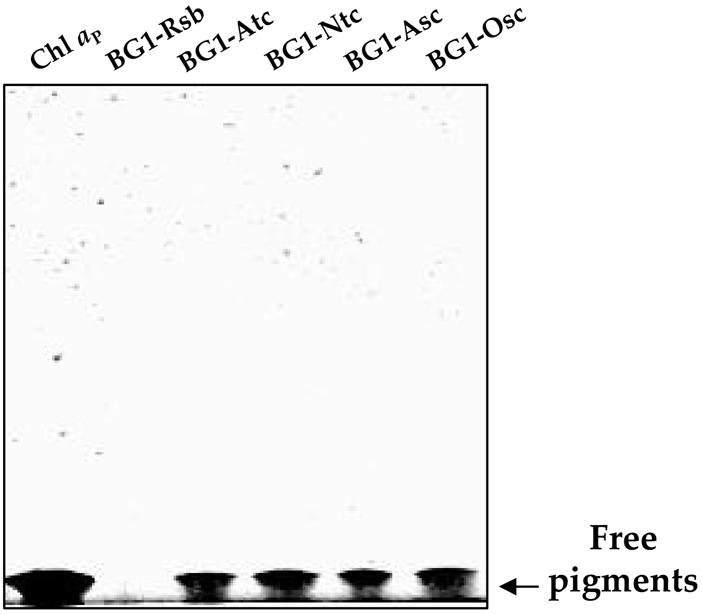

Figure S4. Detection of free Chl a_P in the membranes of BG1 expressing angiosperm *chlG*s. Membranes of BG1 expressing angiosperm *chlGs* were examined for free Chl a_P by CN-PAGE. Free pigments were detected by fluorescence emission under UV-A.

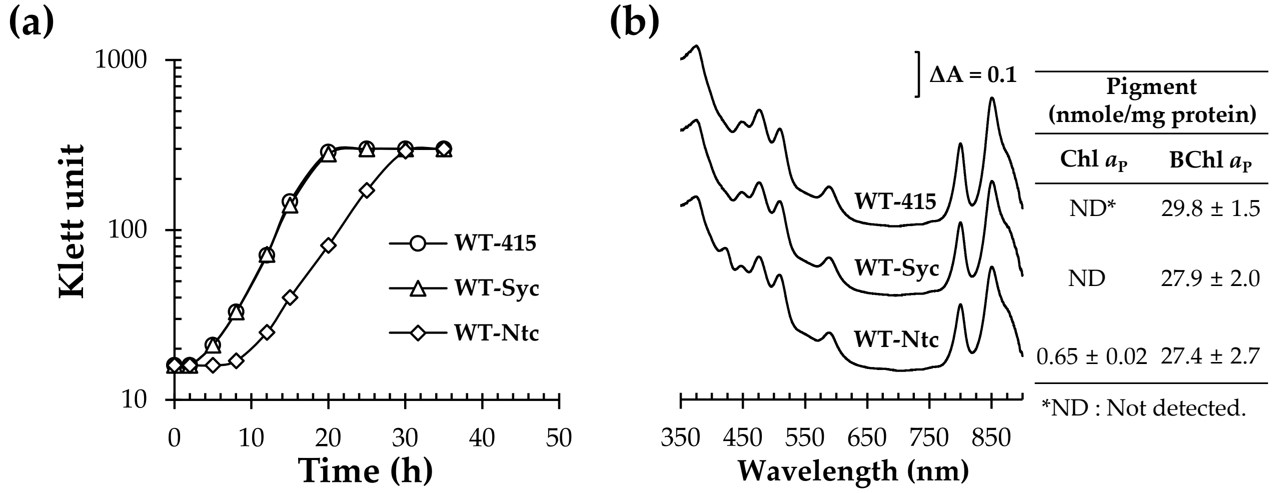

Figure S5. Effect of *NtchlG* expression on the photoheterotrophic growth of WT.
The photoheterotrophic growth (a) and membrane absorption spectrum (b) of WT-Ntc were recorded, in which WT-Syc and WT-415 were used as controls. Pigments were extracted from the membranes and quantified by HPLC, which are listed on the right side of corresponding spectra.

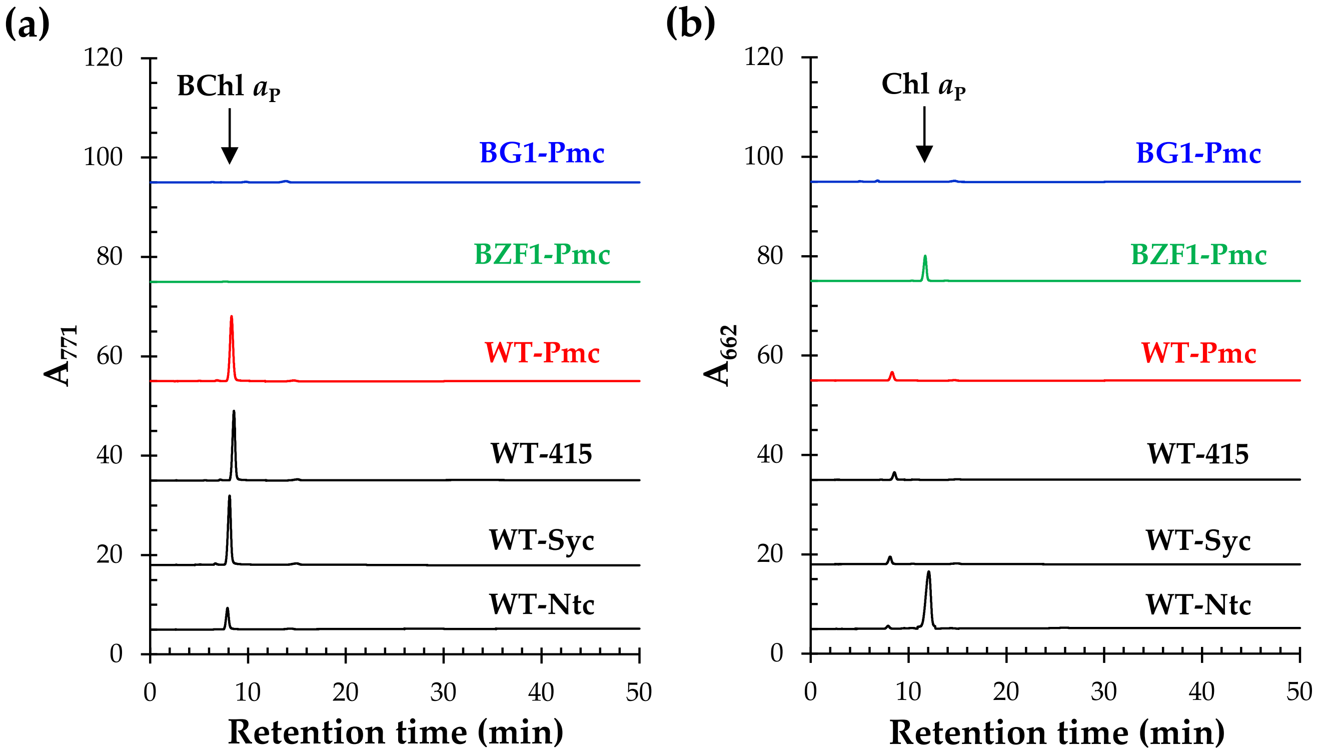

Figure S6. Chl a_P formation by *Prochlorococcus marinus* ChlG in *R. sphaeroides*.
BG1-Pmc and BZF1-Pmc (Table S1) were cultured anaerobically in the dark with 75 mM DMSO for 7 days. WT-Pmc (Table S1) was cultured in semi-aerobic condition for 12 h. WT-415, WT-Syc and WT-Ntc were also grown semi-aerobically and included as controls. Pigments were extracted from whole cells, and analyzed by HPLC to detect BChl a_P (a) and Chl a_P (b). No Chl a_P was formed in BG1-Pmc and WT-Pmc. It was only observed in BZF1 (b), in which metabolic path specific to BChl a_P formation was blocked.

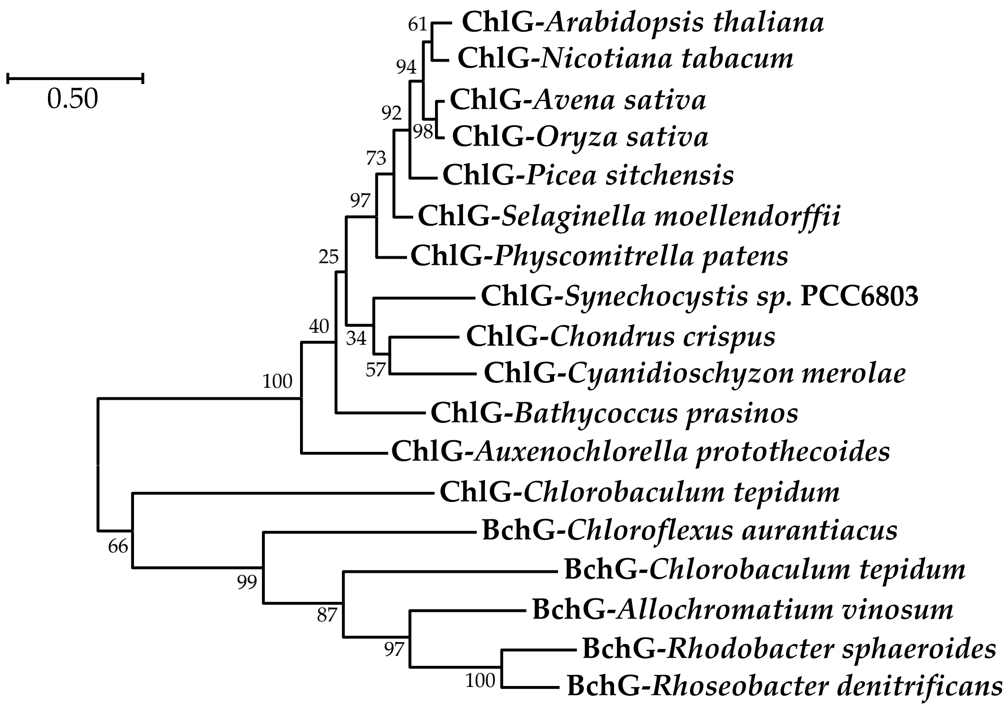

Figure S7. Evolutionary analysis of BchGs and ChlGs.

Evolutionary history was inferred by using the Maximum Likelihood method and Le-Gascuel 2008 model [8]. Tree with the highest log likelihood (-7501.06) is shown. The percentage of trees in which the associated taxa clustered together is shown next to the branches. Initial tree(s) for the heuristic search were obtained automatically by applying Neighbor-Join and BioNJ algorithms to a matrix of pairwise distances estimated using a JTT model, and then selecting the topology with superior log likelihood value. A discrete Gamma distribution was used to model evolutionary rate differences among sites (5 categories (+G, parameter = 0.9867)). The tree is drawn to scale, with branch lengths measured in the number of substitutions per site. This analysis involved amino acid sequence of 13 chlorophyll synthases and 5 bacteriochlorophyll synthases. All positions with less than 95% site coverage were eliminated, i.e., fewer than 5% alignment gaps, missing data, and ambiguous bases were allowed at any position (partial deletion option). There were a total of 287 positions in the final dataset. Evolutionary analyses were conducted in MEGA X [9].

Amino Acid Sequences of BchG and ChlG

1. ChlG-*Arabidopsis thaliana*

MTSILNTVSTIHSSRVTSVDRVGVLSLRNSDSVEFTRRRSGFSTLIYESPGRRFVVRAAETDTDKVKSQTPDKAPAGGSSINQLLGIKGASQETNKWKIRL
QLTKPVTWPPLVWGVVCGAAASGNFHWTPEDVAKSILCMMMSGPCLTGYTQTINDWYDRDIDAINEPYRPIPSGAISEPEVITQVWVLLLGGLGIAG
ILDVWAGHTTPTVFYLALGGSLLSYIYSAPPLKLKQNGWVGNFALGASYISYSIAGLGIAIVNDFKSVEGDRALGLQSLPVAFGTETAKWICVGAIDIT
QLSVAGYLLASGKPYYALALVALIIPQIVFQFKYFLKDPVKYDVKYQASAQPFLVLGIFVTALASQH

2. ChlG-*Nicotiana tabacum*

MASLLNSVPSIKLSNFSNNNPLRSSQISSPFCLSLSRRRLVVRATETDKEVKAQAPDKAPAAGGSSINQILGIKGAKQETDKWKIRVQLTKPVTWPPLV
WGVVCGAAASGNFHWTPEDVAKSVVCMLMSGPFLTGYTQTINDWYDREIDAINEPYRPIPSGAISEQEVINQIWVLLLGGLGLAGILDVWAGHNFP
TIFYLALGGSLLSYIYSAPPLKLKQNGWIGNFALGASYISLPWWAGQALFGTLTPDIIVLTLLYSVAGLGIAIVNDFKSIEGDRAMGLQSLPVAFGSEAA
KWICVGAIDITQISVAGYLLGAGKPYYALALLGLIAPQVFFQFKYFLKDPVKYDVKYQASAQPFLILGLLVTALATSH

3. ChlG2-*Nicotiana tabacum*

MASLLNSVPSIKLSNFSNNNPLRSSQISSPFCLSFSRRRLVVRATETDKEVKAQAPDKAPAAGGSSINQILGIKGAKQETDKWKIRVQLTKPVTWPPLV
WGVVCGAAASGNFHWTPEDVAKSIVCMLMSGPFLTGYTQTINDWYDREIDAINEPYRPIPSGAISGQEVINQIWVLLLGGLGLAGILDVWAGHDFPT
IFYLALGGSLLSYIYSAPPLKLKQNGWIGNFALGASYISLPWWAGQALFGTLTPDIIVLTLLYSVAGLGIAIVNDFKSIEGDRAMRLQSLPVAFGSEAAK
WICVGAIDITQISVAGYLLGAGKPYYALALLGLIAPQVFFQFKYFLKDPVKYDVKYQASAQPFLILGLLVTALATSH

4. ChlG-*Avena sativa*

MATSHPLAAAAATSSSSATFRPPLRFLSSPPSSLTLNRRRSFPVVCAADADAKETTKKPTIPDKAPAAGSSFNQLLGIKGAKQETNIWKIRLQLTKPVT
WPPLVWGVLCGAAASGNFHWTVEDVTKSIVCMLMSGPCLTGYTQTINDWYDRDIDAINEPYRPIPSGAISENEVITQIWVLLLGGLGLGALLDIWAG
HDFPIIFYLALGGSLLSYIYSAPPLKLKQNGWIGNFALGASYIGLPWWAGQALFGTLTPDIVVLTCLYSIAGLGIAIVNDFKSIEGDRTLGLQSLPVAFG
METAKWICVGAIDITQLSVAAYLLSTGKLYYALALLGLTIPQVILQFQYFLKDPVKYDVKYQASAQPFFVFGLLVTALATSH

5. ChlG-*Oryza sativa*

MATSHLLAAASSTAASSATFRPPLLSLRSPPPSSLRLNRRRHFQVVRAAETDKETKANAPEKAPAGGSSFNQLLGIKGAKQENDIWKIRLQLTKPVTW
PPLVWGVLCGAAASGNFHWTVEDVAKSIVCMIMSGPCLTGYTQTINDWYDRDIDAINEPYRPIPSGAISENEVITQIWALLLAGLGLGALLDVWAG
HDFPIIFYLAVGGSLLSYIYSAPPLKLKQNGWIGNFALGASYIGLPWWAGQALFGTLTPDIVVLTSLYSIAGLGIAIVNDFKSVEGDRALGLQSLPVAFG
METAKWICVGAIDITQLSVAGYLFSSGKPYYALALLGLTIPQVVFQFQYFLKDPVKYDVKYQASAQPFFVLGLLVTALATSH

6. ChlG-*Picea sitchensis*

MAALVHGISLTGFKNGSYSGTRSLFKGSYSGTPSLLLRPTPNPILSLHRRNIRVRAAETDTNQVESTSPEKAPAKNEGSSNLNQLLGIKGAAQETDKLKI
RLQLTKPVTWAPLIWGVVCGAASSGNFDWTVEDVTKAIACMVLSGPLLTGYTQTLNDWYDREIDAINEPYRPIPSGAISESEVITQIWVLLLGGLGLA
ALLDVWAGHTVPSVFYIAVGGSLLSYIYSAPPLKLKQNGWIGNFALGSSYICLPWWAGQALFGTLKPDIMVLTVLYSIAGLGIAIINDFKSIEGDRAMG
LQSLPVAFGVDTAKWICVGAIDITQLSIAGYLFATGKQYYALALLGLITPQIILQFQYFLKDPIKYDVKYQASAQPFLVLGLLCTALATSH

7. ChlG-*Selaginella moellendorffii*

MAFLSLYLSKPSTTEALWVSDCRIIHGCCIQMPAARIRFQHRSAGVRSKRHTGRLNVRAAGTETDKTKPNAPEVTPSDTRSSAVNQLLGMKGAGKET
DKWKIRLQLTKPVTWAPLIWGVLCGAAASGNFHWTIEDVAKSITCMLMAGPFLTGYTQTINDWYDREIDAINEPYRPIPSGAISEPEVITQIWVLLLG
GIGLAYTLDVWAGHSFPTIFCLSLGGALLSYIYSAPPLKLKQSGWIGNYALGSSYIALPWWASQALFGTLSWDVVVLTLLYSTAGLGIAIVNDFKSIEGD
RAMGLQSLPVAFGIDTAKWICIGSIDLTQLSVAGYLFATGKLYYALTLLGLITPQIIFQFQYFLKDPLKYDVKYQASAQPFFVLGLLVTALATSH

Amino Acid Sequences of BchG and ChlG

8. ChlG-*Physcomitrella patens*

MVAGGDSKYGSSLTTCEIRFCNCPGLSHSFDNGSGEFSITPLLRLRKILRRPCSHRGSNLGDFKPGRAPVTQLTLQLECVPRDRCDRTSSSHPNQVVGFR
KTGSARTRIILTNYAEHAASPARDYDKADKVSNTPGALRRALQGLSRAGRVPSSSSCRELGVQRSCGESERVVVDAQLHGAHRFSSCFTIPPAPLSRTP
PFTMAMAVMASSAQATARLQLASSNPTKSSVRSLSSCASVAVRGFQPLSLQARGRLSRAGPLRIRAAAKDTEEDKVSKLVDAAPKAAGSAVNQLLGI
KGASAETNKWKIRLQLTKPVTWAPLIWGVLCGAAASGNFQWTLEDVAKSLTCMLMSGPLLTGYTQTINDWYDREIDAINEPYRPIPSGAISEPEVIA
QIWILLLGGLGVAYGLDRWAGHDFPIILCTAIGGSFLSYIYSAPPLKLKQSGWIGNYALGSSYISLPWWAGQALFGTLSWDVVILTLLYSTAGLGIAIVN
DFKSIEGDRQMGLQSLPVAFGIDTAKYICAASIDVTQLAVAAYILYQGKTYYGLGLLALIIPQIILQFKYLLVDPVKYDVKYQASAQPFFVFGLLLTALA
TSH

9. ChlG-*Auxenochlorella protothecoides*

MPAPKISFVPGSRALNLKRVVSVHAKKEGKTTEAEVVPDAPPSKGSAAEDDPNRAFFLGVKGNAQMLGIRGAAVETDIWKIRLQLTKPVTWIPLIWG
VICGAAASGNFEWTFRDCALSALCMLMSGPLLTGFTQTMNDYYDREMDAINEPNRPIPSGAIPEGDVVAQIWILFFTSIAVAYTLDQASPSHDNLQLT
GLALIGSFISFAYSVPWIKLKRSGWIGNYALGSSYIALPWWAGQILFGQLRLDVAILTMLYSIAGLGIAIVNDFKSIKGRVAGEDYVMLPPCNRQRAGA
VWICDRELGLNSLPVAFGIEKAKWICAASIDVTQLGVAAYLAWGLNEPYYAAGIAACLAPQVYAQVRYFLPDPVANDVKYQATAQPFLVIGLLVTA
LAIGHQATPVALG

10. ChlG-*Bathycoccus prasinos*

MASILSVCTTTAAAMNAVVMNASRRSSSLFSSSSSSSSSSSSMRSSSFPGTSLKTSFKSSSKSIKMEQKRRRRHDSGLIVTNMAAGDKVDDLNIKASETNV
SETKLGGDVRQILGFKGASTEDVPVWKIHIQLTKPGTWVPLIWGVMCGAAASGHYEWNLDNIGKALLCMTMSGPFLTGYTQTINDWYDREIDAINE
PYRPIPSGAISETAVKAQIAVLLLGGLACGWQLDQWCEHDFPVIFLLTVFGSWVSYIYSAPPLKLKAEGWKGCYALGSSYIALPWWAGMATFGQLTP
DVMVLTVLYSIAGLGIAIVNDFKSIEGDRALGLQSLPVAFGVEKAKWITVGTIDATQLFVAFYLRGIGEDFYSNVLFCLIAPQIFAQFKFFLPDPIKNDVK
YQAAAQPFLVFGLLATGLAWGHHVNMLAA

11. ChlG-*Chondrus crispus*

MSGSGAPQPPPTEADSPAVKGDSPANDSSDVRQLLGIKGASESTSKWRIRLQLMKPVTWVPLIWGVTCGAAASGNFHWNDPVDIEKSLACMFLAGP
LLTGYTQVINDWYDREIDAINEPYRPIPAGLVTENEVKTQIGVLLAAGLALAAGLDVWAAHEWPVNFTLTVFGVFVAYIYSAPPLKLKQNGWTGSY
ALGASYISLPWWAGQALFGTLDWKTMVLTLFYSFAGLGIAIINDFKSVEGDRATGMQSLPVMFGIDTAKWICVGMIDIFQLLVAGILFGVGEKGYAA
AIVALVAPQMFLQQKYFLKDPVKYDVKYQASAQPFLVIGILVAGLAVGHHGPL

12. ChlG-*Cyanidioschyzon merolae*

MVTPGFISSTVGARGMSSRSCKALALSRIPVLVTWRRTRLNAAKHRGGLRLSQKADDQASPSESKQKSEQSGALDGKQQTTGVNDSEQSLRQLLGMR
GASKETNKWRIRLQLMKPITWIPLVWGVGCGAAASGQYQWNDPQDIAKLLLCMILSGPVLAGFTQTLNDWFDREIDAVNEPYRPIPSGAISEGEVV
AQIWALLFAGLGLAYGLDLWQGHQFPRVLAVALFGTFLAYIYSAPPLKLKKNGWLGNYALGASYISLPWWAGQSLFSDNPLDWKIIALTLLYSFAG
LGIAVINDFKSIEGDRRLGLASLPVMYGVDTAKWLSVGLIDIFQALVAAYLALVGETGYALALVAMILPQVYLQWKIFLPDPMQNDVKYQGAAQPF
LVFGILITALAIGHHGQL

13. ChlG-*Synechocystis* sp. PCC6803

MSDTQNTGQNQAKARQLLGMKGAAPGESSIWKIRLQLMKPITWIPLIWGVVCGAASSGGYIWSVEDFLKALTCMLLSGPLMTGYTQTLNDFYDRDI
DAINEPYRPIPSGAISVPQVVTQILILLVAGIGVAYGLDVWAQHDFPIMMVLTLGGAFVAYIYSAPPLKLKQNGWLGNYALGASYIALPWWAGHALF
GTLNPTIMVLTLIYSLAGLGIAVVNDFKSVEGDRQLGLKSLPVMFGIGTAAWICVIMIDVFQAGIAGYLIYVHQQLYATIVLLLLIPQITFQDMYFLRNP
LENDVKYQASAQPFLVFGMLATGLALGHAGI

Amino Acid Sequences of BchG and ChlG

14. BchG-*Rhodobacter sphaeroides*
MSVNLSLHPRSVPEPRALLELIQPITWFPPIWAYLCGTVSVGIWPGEKWPLVLLGMVLAGPLVCGMSQAANNWCDRHVDAVNEPDRPIPSGRIPGR
WGLYIALLMTVLSLAVGWMLGPWGFGATVFGVLAAWAYSVEPIRLKRSGWWGPGLVALCYEGLPWFTGAAVLSAGAPSFFIVTVALLYAFGAHGI
MTLNDFKALEGDRQHGVRSLPVMLGPEVAAKLACTVMAMAQILVITLLVIWGKPIHAGIITALLVAQLFAMRVLLRDPAGKCPWYNGTGVTLYVL
GMMVAAFAIRGLEVLP

15. BchG-*Allochromatium vinosum*
MNQTTVIATRTYPEPKAILEVLHPITWFPPMWAFTCGVVSSGAPILEQWWLLIAGIILAGPLMCATSQVVNDWYDRDVDAINEPDRPIPSGRIPGRWG
FYLSLIWTVVSLALAYALGPWVFGMALIGMAISWGYSAPPFRFKGNGWWGNLAAGISYEGLAWVTGAAVMIGGALPGWEILVLALLYSLGAHGIM
TLNDFKAIEGDIQMGVRSLPVQLGVEKAAWWACGVMGLPQAVVVALLFSWDKPAHAIAVGVVLLIQVALMVRFLARPVERAVWFSGLGVTVYVT
GMMISAFAVRGLMTAAG

16. BchG-*Chloroflexus aurantiacus*
MSDMSDQTRLSSPPSLPLHKQPQSRYAWLVRSIQLMKPVTWFAPTWAFMCGAIASGALGWNESIGRLLLGMFMAGPILCGLSQVVNDYADREVDAI
NEPHRLIPSGQVSLRHVYILTAVLTWIGASIALFLGRQVAFFVALGLVFALAYSLRPIRGKRNGWIGNALVAISYEGLAWMAGHAAFAPLTGESVTIA
LLYSLGAHGIMTVNDFKSIRGDTIMGIRSIPVQYGKVMAARMVVTTMGVAQIAVIGLLFHWGHPVAATVVAILLAAQSIPNARFIRDPENNEVFFNA
TAIMLYVWGMLAAAIGLAA

17. BchG-*Chlorobaculum tepidum*
MNGSDTLNPELQLNIDEKLHQSGISQSRQKIIRQALENVNRPGFRIEPSAILPLMKPVTWFPPMWAFACGVVSTGESVTDNISILVRGVILAGPLMCAM
SQTMNDYFDREVDAINEPERPIPSGKISKQASWLITFGLILTGFAVALSIHPYVMAIAFVGVLMSHAYSGPPIRAKRNGWFGNLIVGLAYEGVAWLTGS
FAITQGVPSKESIALAIIFSLGAHGIMTLNDFKSVVGDKIRKVASIPVQLGEKNAAILASAVMDIAQIAAIAILVAKGSTIPTAIAVTLLIAQLPMQKILID
HPAEKAVWYNAFGTLLYVLSMMVCAVGIRP

18. ChlG-*Chlorobaculum tepidum*
MALNAKRSAVLPHEVLFLGFTCYFQALSFDQPNTSRVSNNSPDNIPFDTTQERASSGMSRRKPFVQGRRSFEPVSSLALFVRFLKPVTWIPVVWSFICG
AIASGAFGWQQIGEIKFWLAVLLTGPLATGTCQMLNDYFDRDLDEINEPNRPIPGGAISLKSATLLIALWSLLSVVVGWLVHPLIALYVVVGIINAHLY
SANPIKLKKRLWAGNIIVAVSYLIIPWVAGEIAYRSDFSLHAITPSLIVATLYTIASTGTMTINDFKSIEGDRQVGIHTLPAIFGERKAALIAAILIDLGQLM
AAGYMFMIGKAVYGWVTAALVVPQFLLQFSLVRSPRTMDVRYNAIAQNFLVAGMMVCAFAIKSINP

19. BchG-*Rhoseobacter denitrificans*
MAVTDSIHTRRFPEPAAALRLIKPITWFPPMWAYLCGVVSSGVSPVGNWTLVLLGVVLAGPIVCGMSQAANDWCDRHVDAINEPHRPIPSGRIPGR
WGLWIALAMSVLSLGVGWQLGPWGFGATVVGVLAAWAYSAEPVRLKRSGWWGPGLVGLSYETLPWFTGAAVLSAGAPSLPVIVIAVLYGIGAHGI
MTLNDFKALEGDRQMGVNSLPVTLGPQRAAQVACAVMSAPQVVVVFLLFSWGKPLHAAGVCAVLFVQFWAMTIMFKDPKAKAPWYNGTGVLL
YISGMMIAAFALRGLA

20. ChlG-*Prochlorococcus marinus*
MSDTRQLLGIKGGSETTNIWKLRLQLMKPITWIPLLWGVICGAAASGNYHWNLSNFLASLACMVMSGPLLTGYTQTINDYYDREIDAINEPSRPIPSG
AISLTQVRIQIWVLLLLGLSFAYGLDRWAGHSSPSVLYLALGGSLVSYIYSAPPLKLKQNGWLGNYALGASYIALPWWAGQALFGQLTWTTALLTLA
YSLAGLGIAVINDFKSVEGDKKLGLQSLPVVFGIRNASFISAGMIDIFQLAMVGVLILIGQHLASVILILLIIPQITFQDIWLLRDPLEFDVKYQASAQPFLI
LGMLVTALAIGHSSLINI

Construction of Plasmids

1. Plasmid for in-frame *bchP* deletion

A 547-bp DNA upstream from the 60th codon and 499-bp DNA downstream from the 70th codon of *R. sphaeroides bchP* (RSP_0277) were PCR-amplified using primer sets P1F/P1R and P2F/P2R, respectively (All the primers used in this study are listed in Table S2). The DNA fragment from P1F/P1R was digested with *Sph*I/*Eco*RI, and the fragment from P2F/P2R was digested with *Eco*RI/*Xba*I. The resulting fragments were ligated into *Sph*I/*Xba*I sites of pLO1 [6] to form pLO-bchP (Table S3).

2. Plasmids for purification of *A. thaliana* chlorophyllase

A gene fragment of chlorophyllase was PCR-amplified from total cDNA of *Arabidopsis thaliana* using primers P3F/P3R. The fragment was digested with *Bsa*I and cloned into the *Bsa*I site of pASK-IBA3plus (IBA Life sciences, Göttingen, Germany) to yield pChlase (Table S3).

3. Plasmid for expression of water-soluble chlorophyll-binding protein (WSCP) and NtChlG in *R. sphaeroides*

A 698-bp DNA fragment containing *puc* promoter region of *R. sphaeroides* [10] was PCR-amplified using primers P4F/P4R, followed by digestion with *Kpn*I/*Xba*I. A 611-bp DNA fragment coding for WSCP of *Brassica oleracea* and ChlG of *Nicotiana tabacum* (*NtchlG*) were synthesized after optimization on the basis of codon bias of *E. coli* (Bioneer, Daejeon, Korea) as described previously [11]. The WSCP gene fragment was digested with *Xba*I/*Pst*I, and *NtchlG* fragment of pRK-Ntc (Table S3) was digested with *Pst*I/*Eco*RI. Three fragments of *puc* promoter, WSCP gene, and *NtchlG* were ligated together and cloned into the *Kpn*I/*Eco*RI sites of pRK415 [7] to yield pRK-WSCP-Ntc (Table S3).

4. Plasmids for expression of *bchG* and *chlG* in *R. sphaeroides*

A DNA fragment coding for *bchG* (RSP_0279) of *R. sphaeroides* extending from 133-bp upstream from start codon to its stop codon was PCR-amplified using primers P5F/P5R, followed by digestion with *Bam*HI/*Eco*RI. The resulting DNA fragment was cloned into the *Bam*HI/*Eco*RI site of pRK415 to yield pRK-Rsb (Table S3).

A DNA fragment coding for *chlG* (slr0056) of *Synechocystis* sp. PCC6803 extending from 143-bp upstream from start codon to its stop codon was PCR-amplified using primers P6F/P6R, followed by digestion with *Bam*HI/*Eco*RI. The resulting DNA fragment was cloned into the *Bam*HI/*Eco*RI site of pRK415 to yield pRK-Syc (Table S3).

A DNA fragment coding for *NtchlG* was synthesized after codon optimization for expression in *E. coli* (Bioneer, Daejeon, Korea). Ribosomal binding site (RBS; TTTAAGAAGGAGATATACAA) was put at the immediate upstream from start codon. The synthesized DNA fragment was digested with *Xba*I/*Eco*RI and cloned into *Xba*I/*Eco*RI sites of pRK415 to yield pRK-Ntc (Table S3). Likewise, genes coding for other chlorophyll synthases and bacteriochlorophyll synthases were also synthesized with the same RBS after codon optimization for expression in *E. coli*. The synthesized DNA fragments were digested with *Xba*I/*Eco*RI and cloned into *Xba*I/*Eco*RI sites of pRK415 to yield pRK-Atc (*A. thaliana chlG*), pRK-Asc (*Avena sativa chlG*), pRK-Osc (*Oryza sativa chlG*), pRK-Psc (*Picea sitchensis chlG*), pRK-Smc (*Selaginella moellendorffii chlG*), pRK-Ppc (*Physcomitrium patens chlG*), pRK-Apc (*Auxenochlorella protothecoides chlG*), pRK-Bpc (*Bathycoccus prasinos chlG*), pRK-Ccc (*Chondrus crispus chlG*), pRK-Cmc (*Cyanidioschyzon m*erola*e chlG*), pRK-Avb (*Allochromatium vinosum bchG*), pRK-Cab (*Chloroflexus aurantiacus bchG*), pRK-Ctb (*Chlorobaculum tepidum bchG*), pRK-Ctc (*C. tepidum chlG*), pRK-Rdb (*Roseobacter denitrificans bchG*) and pRK-Pmc (*Prochlorococcus marinus chlG*) (Table S3). The amino acid sequences of the cloned ChlGs and BchGs were listed in supplementary materials.

Construction of Plasmids

5. Plasmids for production of BchG and ChlGs in *E. coli*

A DNA fragment coding for *RsbchG* was PCR-amplified from pRK-Rsb using primers P7F/P7R. The resulting DNA was digested with *Nde*I/*Hin*dIII and cloned into the *Nde*I/*Hin*dIII site of pET29a (Novagen, Madison, WI, USA) to generate pET-Rsb (Table S3). Likewise, DNA fragments coding for other chlorophyll synthases were amplified from the corresponding recombinant pRK415 plasmids, digested with *Nde*I/*Hin*dIII, and cloned into the *Nde*I/*Hin*dIII site of pET29a to generate pET-Syc (amplified from pRK-Syc with primers P8F/P8R), pET-Atc (amplified from pRK-Atc with primers P9F/P9R), pET-Ntc (amplified from pRK-Ntc with primers P10F/P10R), pET-Asc (amplified from pRK-Asc with primers P11F/P11R), and pET-Osc (amplified from pRK-Osc with primers P12F/P12R) (Table S3). The gene for iso-enzyme of *N. tabacum* ChlG (*NtchlG2)* extending from start codon to its penultimate codon was synthesized after codon optimization for expression in *E. coli* with flaking enzyme sites of *Nde*I/*Hin*dIII. The resulting DNA fragment harboring *NtchlG2* was digested with *Nde*I/*Hin*dIII and cloned into pET29a to yield pET-Ntc2 (Table S3).

Clear-Native PAGE

Clear-Native polyacrylamide (15%) gel electrophoresis (CN-PAGE) was performed as described previously [12] with some modifications. Membrane fraction was prepared from cells as described earlier in the main text. The membrane fraction was mixed with glycerol at 10% (w/v) prior to loading on polyacrylamide gel. Resolved gel was exposed to UV-A light for detection of free pigments.

Kinetic Analysis of BchG and ChlG Activities in Leaf Extract of *N. tabacum*

ChlG and BchG activities were analyzed with leaf extracts of *N. tabacum*. In order to prevent contamination of any PS bacterium, *N. tabacum* seeds were briefly washed with 75% ethanol containing 0.05% Triton X-100 and dried, which were then planted into sterilized soil and cultivated under fluorescent light (100 μE $m^{-2} s^{-1}$) at 23°C. Plants were grown for 3 weeks after germination in the same condition. Leaves were harvested and frozen in liquid N_2. Ice-chilled Reaction Buffer B (5 ml) was added to 1 g of frozen leaves, and they were immediately ground in precooled mortar, followed by sonication lysis on ice for 5 min for a total of three times. Supernatant (leaf lysate) was decanted after centrifugation at 6,000 ×*g* for 10 min, which was used as an enzyme source for BchG and ChlG assays (1 mg protein for each reaction).

References for Supplementary Materials

1. Sistrom, W.R. A Requirement for Sodium in the Growth of *Rhodopseudomonas spheroides*. *J. Gen. Microbiol.* **1960**, *22*, 778–785, doi:10.1099/00221287-22-3-778.
2. Kim, E.J.; Lee, J.K. Competitive inhibitions of the chlorophyll synthase of *Synechocystis* sp. strain PCC 6803 by bacteriochlorophyllide *a* and the bacteriochlorophyll synthase of *Rhodobacter sphaeroides* by chlorophyllide *a*. *J. Bacteriol.* **2010**, *192*, 198–207, doi:10.1128/JB.01271-09.
3. Kim, E.J.; Kim, J.S.; Lee, I.H.; Rhee, H.J.; Lee, J.K. Superoxide generation by chlorophyllide *a* reductase of *Rhodobacter sphaeroides*. *J. Biol. Chem.* **2008**, *283*, 3718–3730, doi:10.1074/jbc.M707774200.
4. Eraso, J.M.; Kaplan, S. *prrA*, a Putative Response Regulator Involved in Oxygen Regulation of Photosynthesis Gene Expression in *Rhodobacter sphaeroides*. *J. Bacteriol.* **1994**, *176*, 32–43, doi:10.1128/jb.176.1.32-43.1994.
5. Simon, R.; Priefer, U.; Pühler, A. A Broad Host Range Mobilization System for *In Vivo* Genetic Engineering: Transposon Mutagenesis in Gram Negative Bacteria. *Nat. Biotechnol.* **1983**, *1*, 784–791, doi:10.1038/nbt1183-784.
6. Lenz, O.; Schwartz, E.; Dernedde, J.; Eitinger, M.; Friedrich, B. The *Alcaligenes eutrophus* H16 *hoxX* Gene Participates in Hydrogenase Regulation. *J. Bacteriol.* **1994**, *176*, 4385–4393, doi:10.1128/jb.176.14.4385-4393.1994.
7. Keen, N.T.; Tamaki, S.; Kobayashi, D.; Troilinger, D. Improved brood-host-range plasmids for DNA cloning in Gram-negative bacteria. *Gene* **1988**, *70*, 191–197, doi:10.1016/0378-1119(88)90117-5.
8. Le, S.Q.; Gascuel, O. An improved general amino acid replacement matrix. *Mol. Biol. Evol.* **2008**, *25*, 1307–1320, doi:10.1093/molbev/msn067.
9. Kumar, S.; Stecher, G.; Li, M.; Knyaz, C.; Tamura, K. MEGA X: Molecular Evolutionary Genetics Analysis across Computing Platforms. *Mol. Biol. Evol.* **2018**, *35*, 1547–1549, doi:10.1093/molbev/msy096.
10. Lee, J.K.; Kaplan, S. Transcriptional Regulation of *puc* Operon Expression in *Rhodobacter sphaeroides*. *J. Biol. Chem.* **1995**, *270*, 20453–20458, doi:10.1074/jbc.270.35.20453.
11. Hitchcock, A.; Jackson, P.J.; Chidgey, J.W.; Dickman, M.J.; Hunter, C.N.; Canniffe, D.P. Biosynthesis of Chlorophyll *a* in a Purple Bacterial Phototroph and Assembly into a Plant Chlorophyll-Protein Complex. *ACS Synth. Biol.* **2016**, *5*, 948–954, doi:10.1021/acssynbio.6b00069.
12. Wittig, I.; Karas, M.; Schägger, H. High resolution clear native electrophoresis for in-gel functional assays and fluorescence studies of membrane protein complexes. *Mol. Cell. Proteomics* **2007**, *6*, 1215–1225, doi:10.1074/mcp.M700076-MCP200.
